# Genome-wide identification and characterization of TCP transcription factor genes in upland cotton (*Gossypium hirsutum*)

**DOI:** 10.1038/s41598-017-10609-2

**Published:** 2017-08-31

**Authors:** Wen Li, Deng-Di Li, Li-Hong Han, Miao Tao, Qian-Qian Hu, Wen-Ying Wu, Jing-Bo Zhang, Xue-Bao Li, Geng-Qing Huang

**Affiliations:** Hubei Key Laboratory of Genetic Regulation and Integrative Biology, School of Life Sciences, Central China Normal University, Wuhan, 430079 China

## Abstract

TCP proteins are plant-specific transcription factors (TFs), and perform a variety of physiological functions in plant growth and development. In this study, 74 non-redundant *TCP* genes were identified in upland cotton (*Gossypium hirsutum* L.) genome. Cotton TCP family can be classified into two classes (class I and class II) that can be further divided into 11 types (groups) based on their motif composition. Quantitative RT-PCR analysis indicated that *GhTCPs* display different expression patterns in cotton tissues. The majority of these genes are preferentially or specifically expressed in cotton leaves, while some *GhTCP* genes are highly expressed in initiating fibers and/or elongating fibers of cotton. Yeast two-hybrid results indicated that GhTCPs can interact with each other to form homodimers or heterodimers. In addition, GhTCP14a and GhTCP22 can interact with some transcription factors which are involved in fiber development. These results lay solid foundation for further study on the functions of *TCP* genes during cotton fiber development.

## Introduction

TCP proteins, designated from names of four proteins TB1 (TEOSINTE BRANCHED 1) in maize (*Zea mays*), CYC (CYCLOIDEA) in snapdragon (*Antirrhinum majus*) and PCF1/2 (PROLIFERATING CELL FACTORS 1/2) in rice (*Oryza sativa*), are plant-specific transcription factors (TFs). They all contain a highly conserved TCP domain and are widely distributed in higher plants including monocot and dicot species. TCP domain consists of 59 amino acid residues that forms a basic helix-loop-helix (bHLH) type of DNA-binding domain^1^. *Arabidopsis* TCP proteins are classified into two classes, Class I (also named PCF subgroup) and Class II (including CYC/TB1 and CIN subgroups) based on the sequence similarity of the TCP domains^2^. It has been reported that Class I TCPs participate in promoting plant growth and proliferation. While CIN subgroup plays a key role in lateral organ development, and CYC/TB1 subgroup (also named as CYC/DICH) contributes to shoot branching, axillary meristems developing^2^.

TCP proteins usually form homodimers or heterodimers with each other to regulate the target genes’ expression. The target genes of TCP TFs all contain a highly conserved DNA motif G(T/C)GGNCCCAC, especially the core motif TGGGCC, GCCCR, GG(A/T)CCC^3–9^. They can also interact with other TFs such as DELLAs, AS2, ABI4, MYBs, and bHLHs, promoting flavonoid biosynthesis, triggering effector immunity, responding to abiotic stress and mediating salicylic acid (SA), jasmonate (JA), auxin, cytokinin (CK), abscisic acid (ABA) and gibberellin (GA) response^10–21^.

Allotetraploid upland cotton (*G*. *hirsutum*), accounting for more than 90% of cultivated cotton worldwide, is the most important fiber-producing crop^22, 23^. Cotton fibers are the single-cell trichomes derived from epidermal layers of seeds. Fiber development undergoes four distinctive but overlapping developmental stages: initiation (from −2 to 5 day post anthesis, −2–5 DPA), elongation (3–20 DPA), secondary cell wall deposition (16–40 DPA), and maturation (40–50 DPA)^24^. The mechanism of fiber cell differentiation is supposed to be similar to *Arabidopsis* leaf trichome^24–28^. In *Arabidopsis*, the positive regulators, including GL1 (GLABRA1), GL3 (GLABRA3), EGL3 (ENHANCER OF GL3) and TTG1 (TRANSPARENT TESTA GLABRA1), take control over trichome cell fate. GL1 belongs to the R2R3 MYB subfamily, which shows functional redundancy with MYB23 partially. GL3 and its homolog EGL3 are basic helix-loop-helix (bHLH) TFs, while TTG1 is a WD40-repeat protein. These proteins are assembled into a trimeric MYB–bHLH–WD protein complex to promote the expression of *GL2* (encoding a homeodomain/leucine zipper TF) and *TTG2* (encoding a WRKY TF), thereby controlling trichome formation^27, 29^. Similarly, it has been reported that GhMYB2/GhMYB23 (GL1 homolog) and two R2R3 MYBs (GhMYB25 and GhMYB25L), GhDEL65 (GL3 homolog), GhTTG1/GhTTG3 and GhHD1/GhHOX3 (GL2 homolog) regulate fiber initiation and differentiation of cotton^25, 26, 28, 30–32^. Additionally, previous studies showed that overexpressing *GhTCP14* in *Arabidopsis* enhances the initiation and elongation of trichomes by binding to the promoters of auxin-related genes^33^, whereas *GbTCP* (a homolog of *AtTCP15*) silence in cotton leads to shorter fibers, associating with decreased expression of JA biosynthesis genes^34^. These data indicate that *GhTCP14* and *GbTCP* play important roles in fiber development through phytohormone signaling pathways.

Recently, 38 and 36 *TCPs* were identified in two diploid cotton species *Gossypium raimondii* (DD genome) and *Gossypium arboreum* (AA genome), respectively^35, 36^. However, no genome-wide characterization of *TCP* family has been reported in allotetraploid cotton species (such as upland cotton) as so far. On the other hand, genome sequence and annotation of upland cotton (*G*. *hirsutum* TM-1) have been completed recently^22, 23^. This great progress on cotton genome research provides us a great opportunity to identify TCP TFs in the allotetraploid cotton species. In present study, we identified 74 *TCP* genes in upland cotton, and analyzed their gene/protein architectures, conserved domain profiles, physical properties, chromosomal location, and phylogenetic relationship. The expression dynamics of these *TCP* genes in cotton tissues (especially in developing fibers), and the capacity of the cotton TCP proteins to form homodimers/heterodimers, and the interaction with several fiber-related transcription factors were also studied. These data provide valuable information for understanding the classification and putative functions of GhTCPs, also throw some light into further investigation of the molecular mechanism of TCP proteins involved in fiber development.

## Results

### Identification of TCP genes in upland cotton

To identify all members of *TCPs* in upland cotton (*G*. *hirsutum*) genome, we performed a BLASTp search against upland cotton protein database (https://www.cottongen.org/tools/blast/blast) using the TCP sequences of *G*. *raimondii* and *G*. *arboreum* as queries. All potential upland cotton proteins were then submitted to MotifScan and SMART databases for annotation of the domain structure. Only the candidates containing TCP domains were regarded as “true” TCP proteins. Discarding the redundant and partial sequences manually, there are 64 *GhTCPs* in CGP-BGI assembled *Gossypium hirsutum* (AD1) Genome^22^, and 72 *GhTCPs* in NAU-NBI assembled *Gossypium hirsutum* (AD1) Genome^23^. Among all identified *GhTCPs*, 62 members were identical, while the rest 12 *GhTCPs* are different in above two Genome databases through protein sequence alignment. Totally, 74 non-redundant *TCP* genes were identified in upland cotton genome (Table 1). The number of *GhTCPs* is about 3.1 folds of *AtTCPs*, which is slightly higher than the ratio of putative cotton homologs to each *Arabidopsis* gene^22, 23, 37^. Considering upland cotton is an allotetraploid cotton species which contains A and D genomes, we named the 74 putative *TCP* genes as *GhTCP1-A/D* to *GhTCP25-A/D* according to the nomenclature system applied to *Arabidopsis TCPs*.

**Table 1:** TCP gene family in upland cotton (*Gossypium hirsutum* L. acc. TM-1)^a^.

Gene name^b^	Gene symbol	Chromosome and Location	Length (a.a.)	MW (Da)	pI	start and end position of TCP domain	% similarity with AA or DD Genome	Ortholog Gene name and ID in G. arboreum (Length a.a.)	Ortholog Gene name and ID in G. raimondii (Length a.a.)
GhTCP1-A	Gh_A07G1572	A07 58707703–58708935 (−)	410	45.2	9.29	103–161	97.7/97.2	GaTCP1 Cotton_A_09911 (397aa)	GrTCP1 Gorai.001G200400.1 (398aa)
GhTCP1-D	CotAD_68424	Dt_chr1 84695776–84697342(−)	397	43.5	9.18	100–158	98.5/99.2		
GhTCP2-A	Gh_A05G1236	A05 12472085–12473317(+)	410	44.9	7.11	86–144	99.0/96.6	GaTCP2 Cotton_A_26168 (410aa)	GrTCP2 Gorai.009G153900.1 (410aa)
GhTCP2-D	Gh_D05G3838	scaffold4070_D05 17843–19075(+)	410	45.1	8.68	86–144	96.8/98.5		
GhTCP3-A	Gh_A01G0414	A01 6598141–6599481(+)	446	48.4	6.84	37–95	98.9/98.6	GaTCP10 Cotton_A_20110 (448aa)	GrTCP3 Gorai.002G064500.1 (446aa)
GhTCP3-D	Gh_D01G0419	D01 5008710–5010044(+)	444	48.1	6.78	37–95	97.7/98.9		
GhTCP4-A	Gh_A04G0316	A04 7568830–7567625(−)	401	43.8	6.66	38–96	99.5/97.3	GaTCP4 Cotton_A_22289 (401aa)	GrTCP4 Gorai.009G373000.1 (401aa)
GhTCP4-D	Gh_D05G3332	D05 53526717–53527922(+)	401	43.9	6.54	38–96	97.3/99.5		
GhTCP5-A	Gh_A12G1657	A12 78217629–78218606 (+)	325	36.0	6.02	56–114	100/97.8	GaTCP5 Cotton_A_31971 (325aa)	GrTCP5 Gorai.008G199700.1 (327aa)
GhTCP5-D	Gh_D12G1814	D12 50669259–50670242(+)	327	36.3	6.02	56–114	98.8/98.5		
GhTCP6a-A	Gh_A10G0634	A10 10146241–10145336(−)	301	32.0	7.36	57–111	98.0/96.3	GaTCP20b Cotton_A_07501 (298aa)	GrTCP6 Gorai.011G086900.1 (300aa)
GhTCP6a-D	Gh_D10G0762	D10 9085679–9086581(+)	300	31.9	8.62	57–111	98.0/99.3		
GhTCP6b-A	Gh_A05G2936	A05 71576344–71575442(−)	300	31.9	8.17	64–118	99.3/98.0	GaTCP20a Cotton_A_40823 (300aa)	GrTCP20a Gorai.012G084600.1 (300aa)
GhTCP6b-D	Gh_D04G0721	D04 14726091–14725189(−)	300	31.8	8.64	64–118	98.0/100		
GhTCP6c-A	Gh_A09G2496	scaffold2345_A09 21253–22149(+)	298	31.4	9.52	63–117	99.0/98.7	GaTCP20c Cotton_A_39272 (298aa)	GrTCP20b Gorai.006G043800.1 (298aa)
GhTCP6c-D	Gh_D09G0381	D09 13614982–13615878(+)	298	31.5	9.49	63–117	98.0/99.7		
GhTCP7a-A	Gh_A03G1464	A03 94631494–94632270(+)	258	26.9	9.71	35–89	98.8/99.2	GaTCP7a Cotton_A_08973 (258aa)	GrTCP7a Gorai.005G211900.1 257aa
GhTCP7a-D	Gh_D02G1925	D02 62906931–62907704(+)	257	26.7	9.49	35–89	98.1/99.6		
GhTCP7b-A	Gh_A13G0528	A13 12208104–12208871(+)	255	26.4	9.65	34–88	99.6/100	GaTCP21 Cotton_A_26482 (255aa)	GrTCP7b Gorai.013G068600.1 (256aa)
GhTCP7b-D	Gh_D13G0602	D13 8376941–8376171(−)	256	26.5	9.60	34–88	98.4/98.8		
GhTCP8-A	Gh_A04G1120	A04 61394229–61395692(+)	487	51.0	7.73	131–185	99.4/98.2	GaTCP8 Cotton_A_24144 (486aa)	GrTCP8 Gorai.012G166500.1 (488aa)
GhTCP8-D	Gh_D04G1732	D04 49493909–49495372(+)	487	51.0	7.77	131–185	97.1/99.2		
GhTCP9a-A	Gh_A11G0759	A11 7522308–7521292(−)	338	35.4	8.99	74–128	99.4/98.2	GaTCP9a Cotton_A_10947 (338aa)	GrTCP9a Gorai.007G094200.1 (338aa)
GhTCP9a-D	Gh_D11G0887	D11 7686933–7685917(−)	338	35.5	8.99	74–128	97.6/99.4		
GhTCP9b-A	Gh_A12G2051	A12 83425704–83424550(−)	384	41.0	8.74	92–146	98.7/98.2	GaTCP9b Cotton_A_14431 (385aa)	GrTCP19b Gorai.008G243000.1 (388aa)
GhTCP9b-D	Gh_D12G2229	D12 55459140–55457983(−)	385	41.1	8.75	92–146	96.6/98.7		
GhTCP10-A	Gh_A13G1272	A13 66923356–66922127(−)	409	44.2	7.10	37–95	100/98.8	GaTCP3 Cotton_A_23161 (409aa)	GrTCP10 Gorai.013G172800.1 (409aa)
GhTCP10-D	Gh_D13G1576	D13 48232037–48230808(−)	409	44.2	7.12	37–95	99.0/99.3		
GhTCP11-A	Gh_A09G1389	A09 67016702–67017304(+)	200	21.7	8.10	39–93	99.5/98.0	GaTCP11 Cotton_A_24059 (200aa)	GrTCP11 Gorai.006G165300.1 (270aa)
GhTCP11-D	Gh_D09G1394	D09 41442805–41443410(+)	201	21.8	7.78	39–93	99.5/98.0		
GhTCP12-A	Gh_A12G1561	A12 75807605–75809110(+)	501	55.9	7.55	119–177	99.4/98.0	GaTCP12 Cotton_A_37122 (501aa)	GrTCP12 Gorai.008G186800.1 (501aa)
GhTCP12-D	Gh_D12G1689	D12 48768374–48769879(+)	501	55.9	7.15	119–177	98.0/99.0		
GhTCP13a-A	Gh_A05G3219	A05 84247155–84248084(+)	309	34.2	8.80	51–109	100/97.4	GaTCP13a Cotton_A_27227 (309aa)	GrTCP13a Gorai.012G048500.1 (309aa)
GhTCP13a-D	Gh_D04G0387	D04 6071296–6070355(−)	313	34.6	8.58	51–109	97.4/98.7		
GhTCP13b-A	Gh_A09G0084	A09 2154034–2154891(+)	285	32.0	8.17	54–112	100/98.9	GaTCP13b Cotton_A_14726 (285aa)	GrTCP13b Gorai.006G009800.1 (285aa)
GhTCP13b-D	Gh_D09	D09 2175972–2181876(+)	285	32.1	8.17	54–112	95.8/96.8		
GhTCP14a-A	Gh_A11G0279	A11 2574922–2576109(−)	395	42.3	7.25	98–152	99.2/99.2	GaTCP14a Cotton_A_09220 (395aa)	GrTCP14a Gorai.007G036800.1 (395aa)
GhTCP14a-D	Gh_D11G0333	D11 2835253–2836440(−)	395	42.2	7.39	98–152	99.5/99.5		
GhTCP14b-A	Gh_A07G0574	A07 7929041–7930297(+)	418	44.5	8.84	96–150	98.8/96.3	GaTCP14b Cotton_A_02703 (418aa)	GrTCP14b Gorai.001G072200.1 (409aa)
GhTCP14b-D	Gh_D07G0639	D07 7418297–7419526(+)	409	43.1	8.49	87–141	96.1/99.3		
GhTCP14c-A	Gh_A12G1603	A12 76852216–76850996(−)	406	44.1	7.21	91–145	98.3/97.0	GaTCP14c Cotton_A_27685 (406aa)	GrTCP14c Gorai.008G192400.1 (401aa)
GhTCP14c-D	Gh_D12G1742	D12 49672741–49671539(−)	400	43.3	6.84	85–139	96.5/98.8		
GhTCP15a-A	Gh_A12G1522	A12 74801349–74802383(+)	344	37.6	8.44	49–103	99.1/97.4	GaTCP15a Cotton_A_06142 (342aa)	GrTCP15a Gorai.008G181600.1 (344aa)
GhTCP15a-D	Gh_D12G1644	D12 47951010–47952044(+)	344	37.6	8.75	49–103	98.0/99.4		
GhTCP15b-A	Gh_A13G0648	A13 18142453–18141353(−)	366	39.7	9.42	51–105	98.4/96.2	GaTCP15b Cotton_A_33342 (365aa)	GrTCP15b Gorai.N023400.1 (365aa)
GhTCP15b-D	Gh_D13G2530	scaffold4706_D13 49814–48717(−)	365	39.7	8.66	53–107	95.9/99.2		
GhTCP15c-A	Gh_A13G0647	A13 18135152–18136204(−)	350	38.0	9.55	53–107	99.4/97.1	GaTCP15c Cotton_A_33341 (352aa)	GrTCP15c Gorai.013G084500.1 (352aa)
GhTCP15c-D	Gh_D13G2529	scaffold4706_D13 42955–44010(−)	351	38.1	9.20	51–105	96.8/99.7		
GhTCP16-A	Gh_A13G2021	A13 79662978–79662388(−)	196	21.1	8.78	40–94	99.5/98.0	GaTCP16 Cotton_A_10509 (196aa)	GrTCP21 Gorai.013G268200.1 (196aa)
GhTCP16-D	Gh_D13G2419	D13 60228700–60228110(−)	196	21.1	8.80	40–94	98.0/99.0		
GhTCP17-A	Gh_A07G0613	A07 8581877–8581005(−)	266	30.2	7.88	45–103	99.6/98.5	GaTCP17 Cotton_A_19125 (266aa)	GrTCP17 Gorai.001G076700.1 (266aa)
GhTCP17-D	Gh_D07G0680	D07 8039581–8038708(−)	266	30.3	7.88	45–103	99.2/99.6		
GhTCP18a-A	Gh_A11G0057	A11 566289–570021(+)	329	37.8	9.10	112–170	99.4/93.8	GaTCP18a Cotton_A_07573 (329aa)	GrTCP18a Gorai.007G007500.1 (324aa)
GhTCP18a-D	Gh_D11G0061	D11 570277–571355(+)	328	37.6	8.74	110–168	95.4/97.2		
GhTCP18b-A	Gh_A12G2405	A12 86543026–86541847(−)	367	41.6	8.78	121–179	95.1/96.4	GaTCP18b Cotton_A_01394 (367aa)	GrTCP18b Gorai.008G285300.1 (361aa)
GhTCP18b-D	Gh_D12G2641	scaffold4574_D12 6298–5108(−)	361	40.8	8.08	121–179	91.3/98.9		
GhTCP19a-A	Gh_A09G1605	A09 69634296–69633271(−)	341	36.9	6.55	89–143	99.1/98.5	GaTCP19a Cotton_A_21588 (341aa)	GrTCP19a Gorai.006G197000.1 (337aa)
GhTCP19a-D	Gh_D09G1703	D09 44763016–44764029(−)	337	36.4	6.62	89–143	98.2/98.5		
GhTCP19b-A	Gh_A08G1602	A08 94661478–94660444(−)	312	34.0	9.60	61–122	92.8/88.1	GaTCP19b Cotton_A_09964 (335aa)	GrTCP9b Gorai.004G206900.1 (345aa)
GhTCP19b-D	Gh_D08G1913	D08 57072639–57071603(−)	322	33.7	8.27	61–122	92.6/98.4		
GhTCP20a-A	Gh_A07G2121	A07 77801046–77801960(+)	279	29.7	9.07	33–87	99.7/97.7	GaTCP20d Cotton_A_22689 (306aa)	GrTCP20c Gorai.001G273300.1 (300aa)
GhTCP20a-D	Gh_D07G2330	D07:54858081.0.54858995(+)	304	32.6	8.63	58–112	97.7/99.0		
GhTCP20b-A	Gh_A12G1302	A12 69066765–69065875(−)	296	31.5	9.64	64–118	98.8/97.6	GaTCP6 Cotton_A_23025 (255aa)	GrTCP20d Gorai.008G157300.1 (298aa)
GhTCP20b-D	Gh_D12G1425	D12 43870949–43870059(−)	296	31.6	9.64	64–118	97.6/100		
GhTCP21-A	Gh_A12G1214	A12 66186677–66185946(−)	243	25.3	9.91	34–88	97.9/97.9	GaTCP7b Cotton_A_14593 (243aa)	GrTCP7c Gorai.008G147800.1 (243aa)
GhTCP21-D	Gh_D12G1337	D12 41835395–41834664(−)	243	25.4	10.0	34–88	97.5/99.2		
GhTCP22-A	Gh_A01G1534	A01 91500055–91501707(+)	550	57.9	6.96	176–230	99.5/98.7	GaTCP22 Cotton_A_27060 (553aa)	GrTCP22 Gorai.002G215000.1 (549aa)
GhTCP22-D	Gh_D01G1783	D01 54880882–54882528(+)	548	57.7	7.03	174–228	98.7/99.5		
GhTCP23-A	Gh_A05G2343	A05 28649369–28648101(−)	422	44.8	7.06	103–157	98.6/97.4	GaTCP23 Cotton_A_03998 (418aa)	GrTCP23 Gorai.009G289000.1 (421aa)
GhTCP23-D	Gh_D05G2610	D05 26954135–26952870(−)	421	44.7	7.25	102–156	97.8/99.0		
GhTCP24-A	Gh_A10G0394	A10 3904862–3903471(−)	463	50.1	7.36	85–143	99.4/99.6	GaTCP24 Cotton_A_02913 (463aa)	GrTCP24 Gorai.011G046000.1 (463aa)
GhTCP24-D	CotAD_26716	D10 3715141–3713750(−)	463	50.1	7.36	85–143	99.4/99.6		
GhTCP25-A	Gh_A04G0489	A04 25693571–25695720(+)	405	50.3	6.67	46–100	96.3/86.9	GaTCP25 Cotton_A_37650 (431aa)	GrTCP25 Gorai.009G398700.1 (435aa)
GhTCP25-D	Gh_D04G0925	D04 27075384–27077529(+)	436	48.4	6.28	46–100	88.9/99.8		

^a^Genes information in G. hirsutum from Zhang *et al*. (2015).

^b^A and D were derived from the A-genome and D-genome progenitor in the tetraploid cotton.

The CotAD_68424 and CotAD_26716 was named by Institute of Cotton Research of the Chinese Academy of Agricultural Sciences, Anyang, China and sequenced by BGI-Shenzhen, Shenzhen, China. The other “gene symbol” was named by Nanjing Agricultural University, Nanjing, China and sequenced by Novogene Bioinformatics Institute (NBI), Beijing, China.

### Phylogenetic relationship of the cotton TCP family

**Figure 1:**
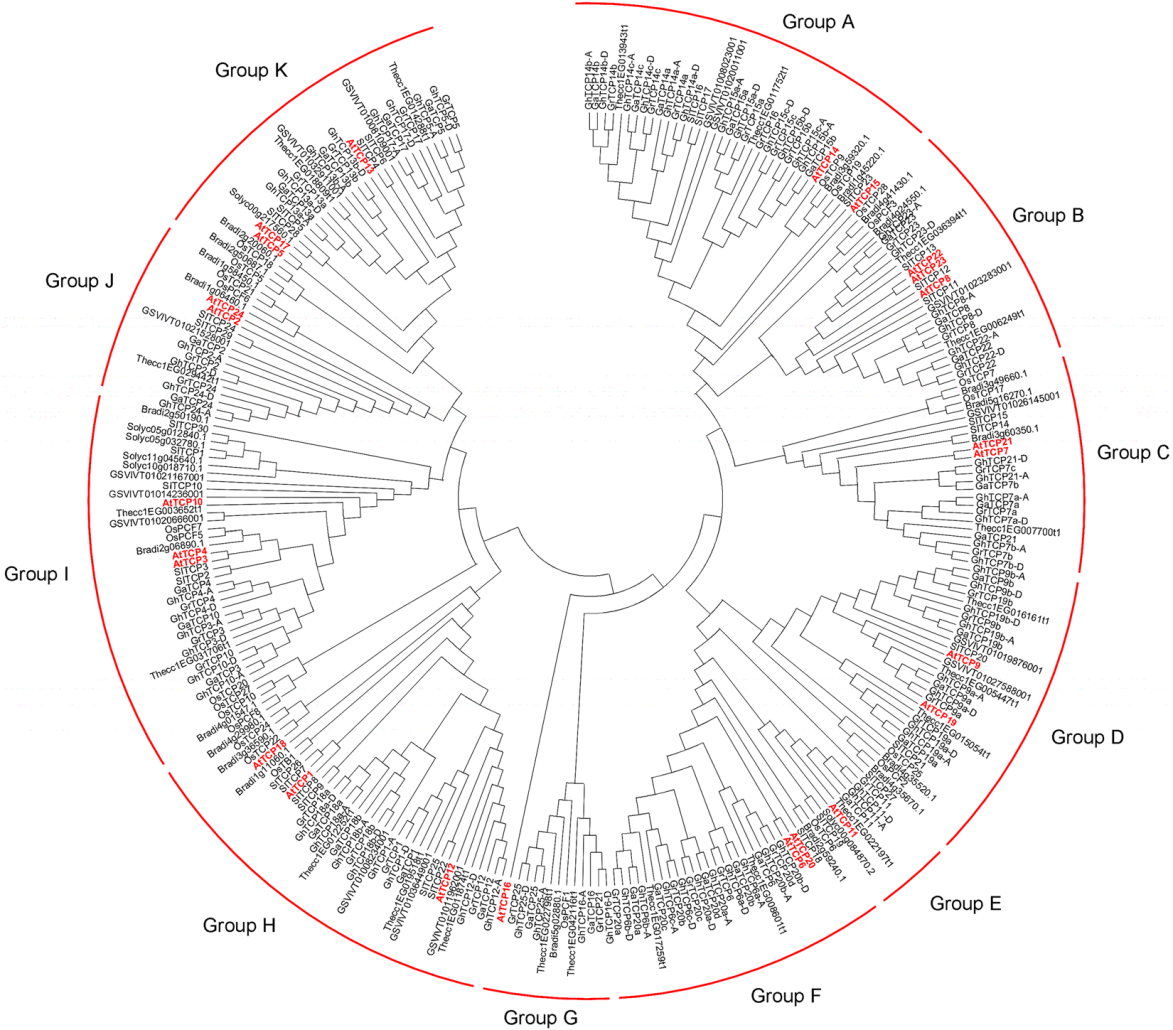
Phylogenetic analysis of upland cotton (*G*. *hirsutum*) TCP family. Phylogenetic tree was constructed using 298 protein sequences from *G*. *hirsutum* A-subgenome (37) and D-subgenome (37), *G*. *arboreum* (36), *G*. *raimondii* (38), *Arabidopsis thaliana* (24), *Solanum lycopersicum* (36), *Oryza sativa* (23), *Brachypodium distachyon* (21), *Theobroma cacao* (31), and *Vitis vinifera* (15) by Neighbor-joining method in MEGA 6.06 with bootstrap replication of 1000 times. *Arabidopsis* TCPs are highlighted with red colored text.

To reveal the evolutionary relationship of the identified cotton TCP proteins, a phylogenetic tree was constructed by Neiboring-Joining (NJ) method using the full length 298 TCP protein sequences from *G*. *hirsutum, G*. *arboreum*, *G*. *raimondii*, *Theobroma cacao*, *Vitis vinifera*, *Arabidopsis thaliana*, *Solanum lycopersicum*, *Oryza sativa*, and *Brachypodium distachyon*. As shown in Fig. 1, the TCP family is divided into 11 groups designated Group A to Group K. GhTCPs in Group A−G belong to PCF clade, while Group H belongs to CYC/TB1 clade and Group I−K belong to CIN clade (Table 2)^2, 35, 36^. Group A, the largest clade among all groups, contains 12 *GhTCP* members, accounting for 16.2% of total *GhTCPs*; Group E, the smallest clade, only contains 2 members. Out of the 74 *GhTCPs*, 48 members belong to class I and the rest 26 fall into class II. In *Arabidopsis*, there are 13 class I *TCPs* and 11 class II *TCPs*. Compared with *Arabidopsis TCPs*, the expansion of *TCPs* in *G*. *hirsutum* genome is biased, which occurs mainly in class I (about 3.7 folds expansion). The class II remains about 2.5-fold size as that in *Arabidopsis* (Fig. 1, Table 2). In addition, we found that Group E is specific for eudicots species. And among the eight chosen species, only *Vitis vinifera* lacks Group E, F, G. This may imply that the divergence of these species took place after the *TCP* transcription factor family expansion.

**Table 2:** Number of *TCPs* in upland cotton (*G*. *hirsutum*), *G*. *arboreum*, *G*. *raimondii*, *Arabidopsis thaliana*, *Solanum lycopersicum*, *Oryza sativa*, *Brachypodium distachyon*, *Theobroma cacao*, and *Vitis vinifera*.

Species	Class I (PCF, Group A-G)	Class II (CYC/TB1, Group H)	Class II (CIN, Group I-K)	Total
*G*. *hirsutum*	48	8	18	74
*G*. *arboreum*	23	4	9	36
*G*. *raimondii*	25	4	9	38
*Arabidopsis*	13	3	8	24
*Solanum lycopersicum*	14	6	16	36
*Oryza sativa*	10	3	10	23
*Brachypodium distachyon*	11	3	7	21
*Theobroma cacao*	13	3	5	21
*Vitis vinifera*	6	3	6	15

### Chromosomal distribution and gene duplication

**Figure 2:**
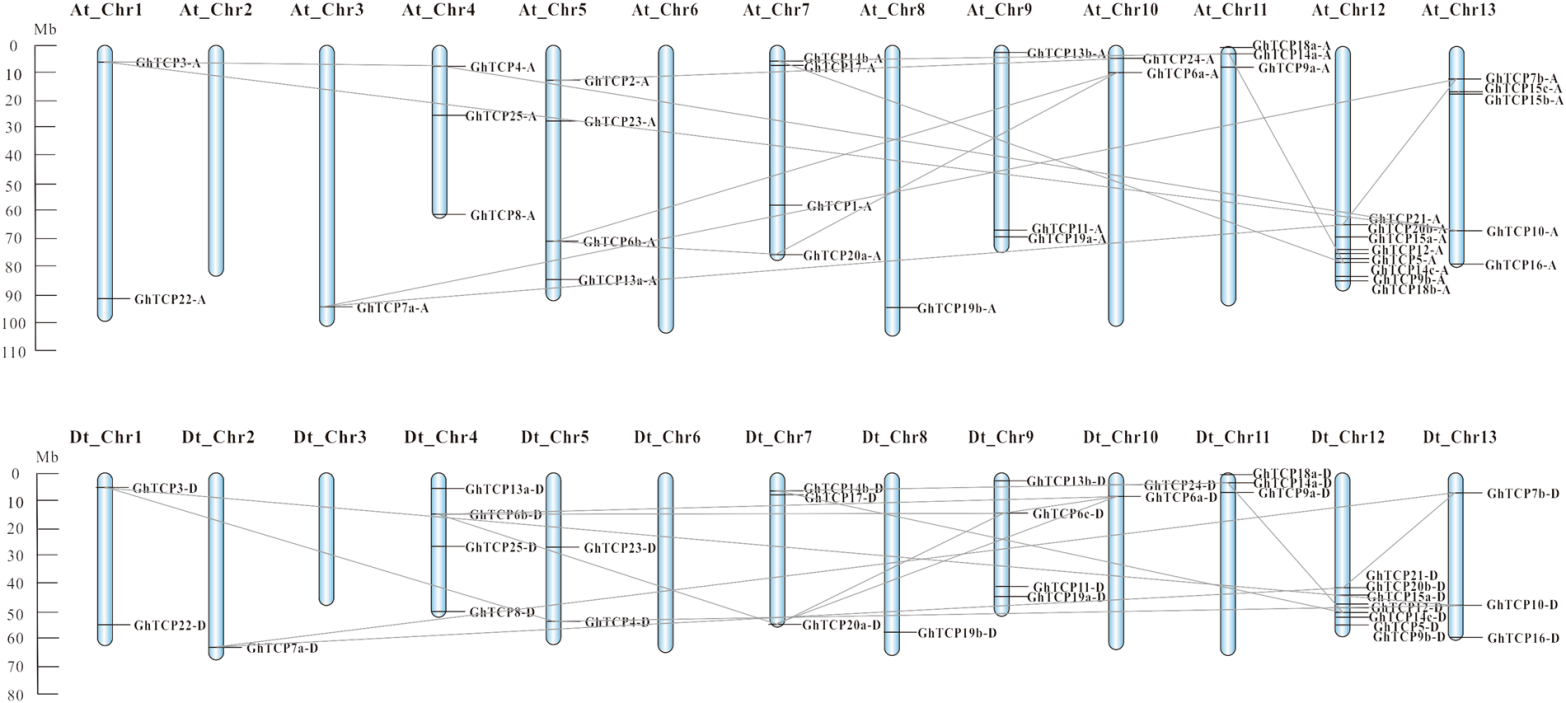
Physical locations and gene duplication status of TCP genes on upland cotton (*G*. *hirsutum*) chromosomes. The *TCP* genes are located according to the upland cotton (*G*. *hirsutum*) genome NAU-NBI Assembly V 1.1 and Annotation v1.1 in COTTONGEN (https://www.cottongen.org/find/genes), and possible gene duplication events are indicated by gray lines.

Among the 74 *GhTCPs*, 69 members are located at the 22 chromosomes, and the else five genes are located in 4 unmapped scaffolds (scaffold4574_D12, scaffold4706_D13, scaffold2345_A09, and scaffold4070_D05). The distribution of *GhTCP* genes on the chromosomes is uneven, with the number of *TCP* genes per chromosome ranging from 0 to 7. Chromosomes At_Chr12 and Dt_Chr12 contain seven genes, while no *TCP* gene is found on At_Chr2, Dt_Chr3, At_Chr6 and Dt_Chr6 (Fig. 2). The distribution patterns of *TCP* genes in *G*. *hirsutum* chromosomes are similar to that in *G*. *raimondii*, but more uneven than that in *G*. *arboreum* ^35, 36^.

Additionally, the gene duplication events were further investigated to reveal the expansion mechanism of the *TCP* gene family in *G*. *hirsutum*. As shown in Fig. 2, 14 pairs of duplicated genes in A-genome and 15 pairs of duplicated genes in D-genome were identified, accounting for about 70% of cotton *TCP* gene family. In fact, as the five genes located in unmapped scaffolds also show high identity to other genes, there could be even more duplication events. Further, except *GhTCP15b* and *GhTCP15c*, all the paralogous gene pairs are located on different chromosomes, suggesting that they result from segment duplications rather than tandem duplications.

### Genomic structure of *GhTCP* genes and domain analysis of their protein products

**Figure 3:**
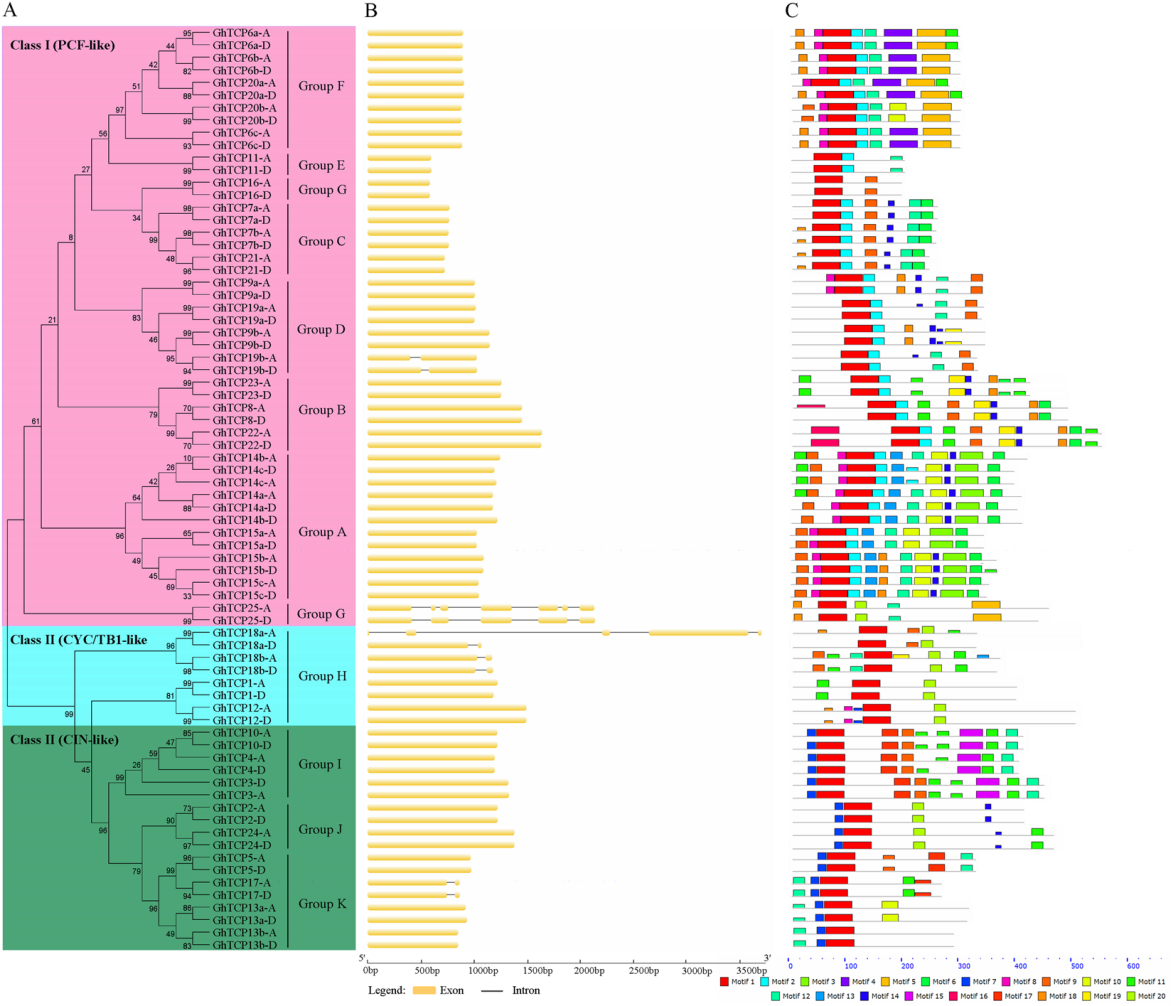
Characterization of upland cotton (*G*. *hirsutum*) TCPs. (**A**) Phylogenetic analysis of GhTCP proteins. The phylogenetic tree was generated using the Neighbor-Joining (NJ) method implemented in the MEGA 6.0 software with JTT model and pairwise gap deletion option. The bootstrap analysis was conducted with 1000 iterations. (**B**) exon/intron organization of *GhTCP* genes. Exons and introns are indicated with yellow boxes and gray lines, respectively. (**C**) Motif composition of GhTCP proteins. Conserved motifs in the GhTCP proteins are indicated by colored boxes.

To get a better understanding of the diversification of the *GhTCP* genes, the exon/intron organization of *GhTCPs* were analyzed. As shown in Fig. 3B, most (64 out of 74) of *GhTCP* genes contain no intron, and 7 members contain only one intron in the open reading frame (ORF). However, two genes (*GhTCP18a-A and GhTCP25-D*) consist of four introns and five exons, and one gene (*GhTCP25-A*) possesses six introns and seven exons. Moreover, similar exon/intron structures were found in *GhTCP* genes within the same phylogenetic subfamily (Fig. 3B).

To further reveal the diversification of cotton TCP family, putative motifs of cotton TCP proteins were predicted by program MEME choosing 20 motifs’ mode (Fig. 3C, Supplementary Fig. S1, and Supplementary Table 1). Based on the composition of motifs, the GhTCP proteins can be classified into 11 groups, just the same as that in Figs 1 and 3A,C). Motif 1 was identified as the conserved TCP domain which is present in every *G*. *hirsutum* TCP protein, providing further support for the reliability of our identification (Fig. 3C, Supplementary Fig. S1, and Supplementary Table 1). GhTCPs members within a sub-clade usually exhibit similar motif composition, while the motif composition among GhTCPs members from distinct clades shows significant difference, It indicates that there is possible intra-subclade functional redundancy and inter-subclade functional divergence (Fig. 3C).

### Expression profiling of TCP genes in cotton

**Figure 4:**
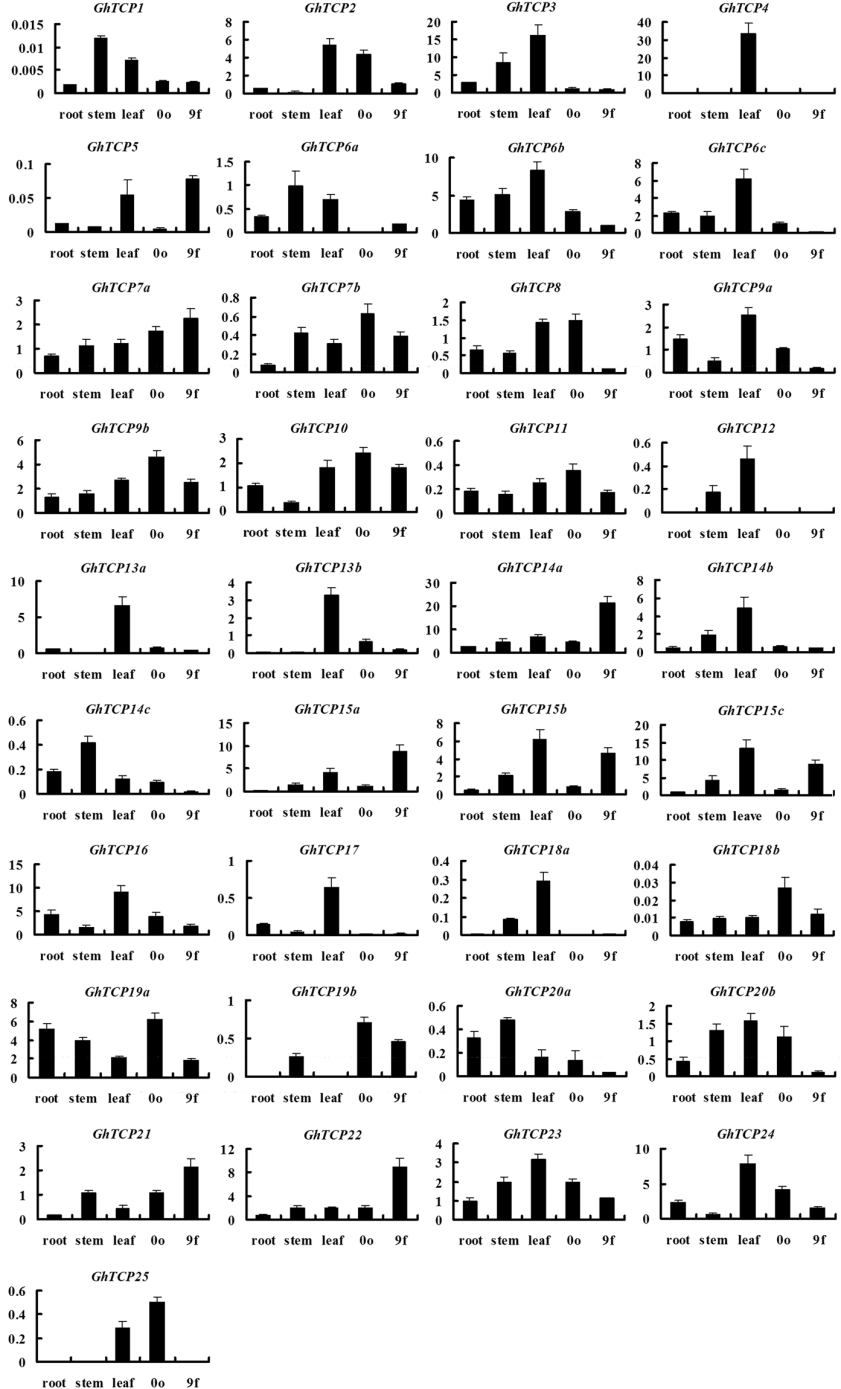
Quantitative RT-PCR analysis of expressions of *TCP* genes in upland cotton tissues. 0o and 9 f indicate 0 DPA (day post anthesis) ovules and 9 DPA fibers, respectively. Error bars indicate ± SD of triplicate experiments. Three biological replicates were used for calculation. Y-axis represents the relative expression value (%) to *GhUBI1* gene.

To investigate the functional divergence of cotton *TCP* genes, their expression levels in different organs/tissues (including roots, stems, leaves, ovules and fibers) were analyzed by quantitative RT-PCR (qRT-PCR). Because of the high sequence similarity between *GhTCP-A* and *GhTCP-D* cDNAs, we designed one common primer pair for analyzing *TCP-A/D* gene expression. As shown in Fig. 4, the expression of *GhTCP7a*, *GhTCP9b*, *GhTCP11*, *GhTCP19a* and *GhTCP23* showed no tissue difference, with relatively high expression levels in all tissues. The majority of the rest genes’ expression exhibit obvious tissue difference. For example, *GhTCP2*, *GhTCP3*, *GhTCP4*, *GhTCP5*, *GhTCP6a/6b/6c*, *GhTCP7a/7b*, *GhTCP9a/9b*, *GhTCP10*, *GhTCP11*, *GhTCP12*, *GhTCP13a/13b*, *GhTCP14b*, *GhTCP15b/15c*, *GhTCP16*, *GhTCP17*, *GhTCP18a*, *GhTCP20b*, *GhTCP23* and *GhTCP24* were specifically or preferentially expressed in leaves. These genes are homologs of class I and *CIN AtTCPs* which are involved in regulating leaf morphology^4, 38–43^. This indicates that these genes may be associated with developmental regulation of cotton leaves. The transcripts of some other genes, such as *GhTCP1*, *GhTCP6a*, *GhTCP14c* and *GhTCP20a*, were predominantly accumulated in stems. The different expression patterns of *GhTCPs* in cotton suggest the functional divergence of these *GhTCP* genes in cotton development.

**Figure 5:**
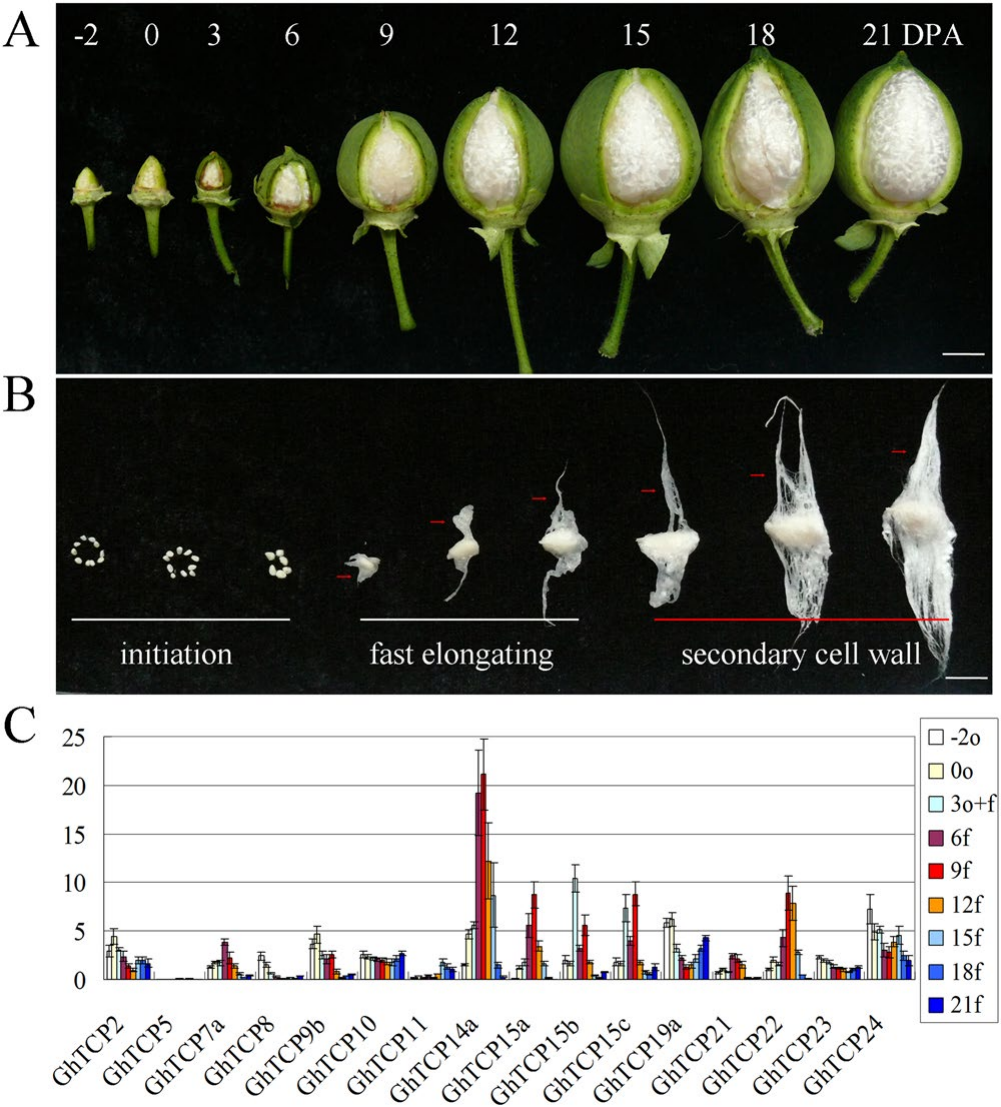
Quantitative RT-PCR analysis of Epressions of *GhTCP* genes in developing fibers. (**A**) Cotton boll and fiber development: bolls at increasing stages of development were partially dissected to show ovules. (**B**) Cotton fiber development is shown over developmental time. Red arrow showed the fiber cells. All scale bars = 1 cm. (**C**) Epressions of *GhTCP* genes in developing fibers. Relative values of expressions of *GhTCP* genes in fibers are shown as percentage of *GhUBI1* expression activity. Error bars represent SD. −2o and 0o represent −2 and 0 DPA ovules; 3o + f represents 3DPA ovules with fibers; 6f–21 f represent 6DPA fibers to 21 DPA fibers. Error bars indicate ± SD of triplicate experiments. Three biological replicates were used for calculation. DPA, day post anthesis. Y-axis represents the relative expression value (%) to *GhUBI1* gene.

We are more concerned about the function of the *TCP* genes in fiber development. qRT-PCR results showed that *GhTCP2*, *GhTCP7a/7b*, *GhTCP8*, *GhTCP9b*, *GhTCP10*, *GhTCP11*, *GhTCP19a/19b*, *GhTCP20b*, *GhTCP23* and *GhTCP24* were strongly expressed in 0 DPA ovules relatively. While *GhTCP5*, *GhTCP7a*, *GhTCP9b*, *GhTCP10*, *GhTCP14a*, *GhTCP15a*/*15b*/*15c*, *GhTCP19b*, *GhTCP21* and *GhTCP*22 were expressed in 9 DPA fibers at relatively high levels. The 0 DPA ovules and 9 DPA fibers refer to the cotton fiber cells at the stages of initiation and fast elongation, respectively. Therefore, some genes, which are relatively higher expressed in 0 DPA ovules or 9 DPA fibers, were selected out as candidates to investigate their expression patterns during cotton fiber development. As shown in Fig. 5C, Class I members, including *GhTCP7a*, *GhTCP14a*, *GhTCP15a/15b/15c*, *GhTCP21* and *GhTCP22*, were preferentially expressed in fast elongating fibers (6~12 DPA), especially, Group A members (*GhTCP14a* and *GhTCP15a/15b/15c*) which were predominantly expressed in the fibers of this stage (Fig. 5C). The result implied that Class I, especially Group A, *TCP* genes may be involved in cotton fiber elongation. *GhTCP2*, *GhTCP8*, *GhTCP9b*, *GhTCP19a*, *GhTCP23* and *GhTCP24* were preferentially expressed at the stage of fiber initiation. Relatively, *GhTCP2*, *GhTCP10*, *GhTCP11*, *GhTCP19a* and *GhTCP24* were highly expressed in secondary cell wall deposition stage (Fig. 5C). Furthermore, expression patterns of these genes were verified by using transcriptome data during cotton fiber development. The RPKM (reads per kb per million reads) values denoting the expression levels of *TCP* genes in the cotton -3, 0, 3 DPA ovule, 5, 10, 20, and 25 DPA fibers were used to create a heat-map of *TCP* expression (Table S2). As shown in Supplementary Fig. S2, *GhTCP7a*, *GhTCP14a*, *GhTCP15a/15b/15c*, *GhTCP20b*, *GhTCP21-D*, *GhTCP22* and *GhTCP25-A* were preferentially expressed in fast elongating fibers. *GhTCP1-A*, *GhTCP3*, *GhTCP4-D*, *GhTCP5*, *GhTCP6a/6b/6c*, *GhTCP10*, *GhTCP11*, *GhTCP12-D*, *GhTCP13a* and *GhTCP20a-D* were preferentially expressed in secondary cell wall deposition stage. *GhTCP2*, *GhTCP7b*, *GhTCP8*, *GhTCP9a/9b*, *GhTCP14b/14c*, *GhTCP12-A*, *GhTCP16*, *GhTCP19a/19b*, *GhTCP20a-A*, *GhTCP23*, *GhTCP24-A* and *GhTCP25-D* were preferentially expressed in cotton fiber initiation. The transcriptome data were consistent with the qRT-PCR results (Fig. 5C, Supplementary Fig. S2). These results suggest that *GhTCPs’* expression is developmentally regulated in cotton fibers.

### Differential expressions of *GhTCPs* in cotton Xuzhou 142 and its natural fuzzless-lintless mutant (*fl*)

**Figure 6:**
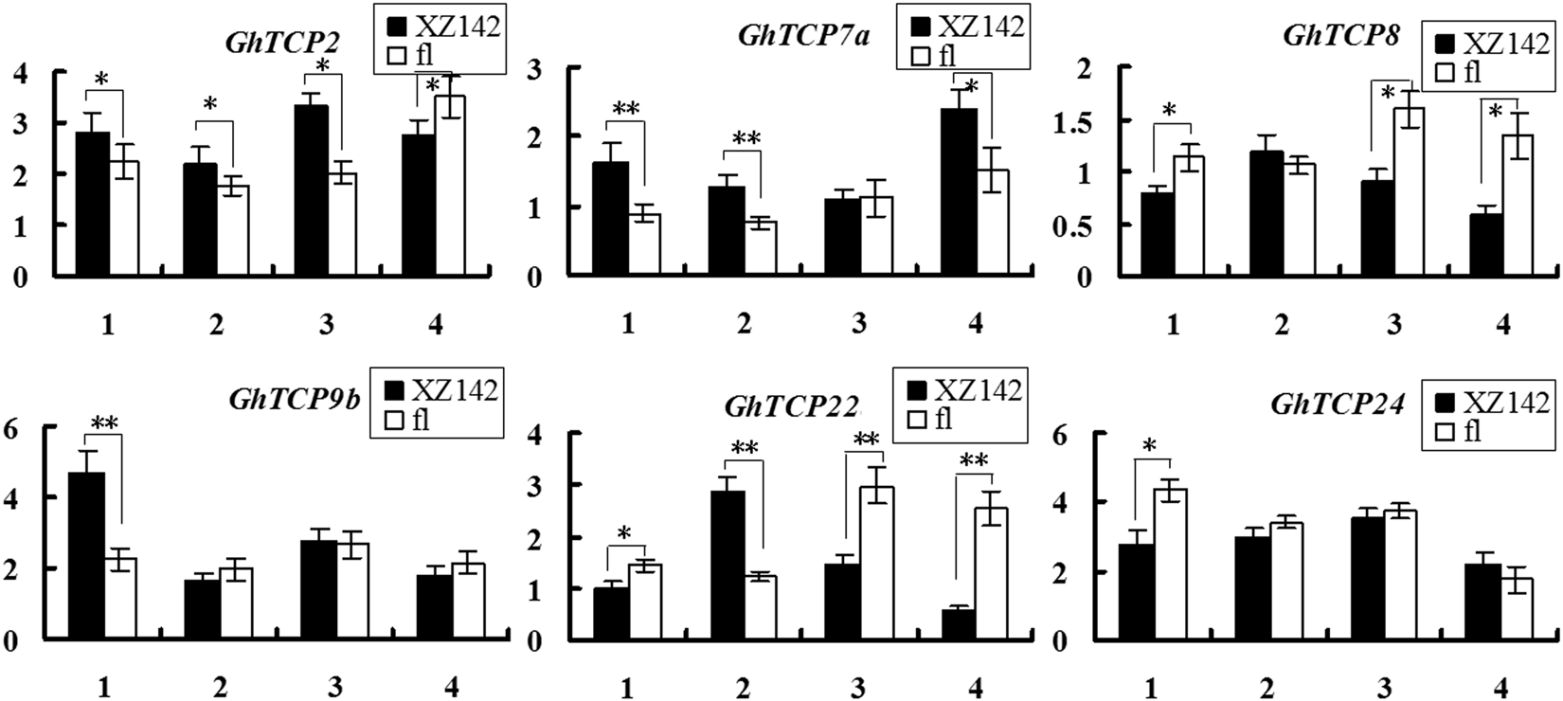
Comparison of expressions of *GhTCP* genes in upland cotton XuZhou142 and its fiberless mutant (*fl*). Quantitative RT-PCR was performed for analyzing expression levels of TCP genes in early developing ovules of wild type cotton Xuzhou 142 and *fl*. 1,2,3,4 represent the cotton ovules at −2, −1, 0 and 1 DPA (day post anthesis), respectively. Error bars indicate ± SD. Three biological replicates were used for calculation. *. There was significant difference in gene expression level between Xuzhou 142 and *fl* (P < 0.05). **. There was very significant difference in gene expression level between Xuzhou 142 and *fl* (P < 0.01). Y-axis represents the relative expression value (%) to *GhUBI1* gene.

To determine whether *GhTCPs* are involved in fiber initiation, we analyzed the expressions of six *GhTCP* genes (*GhTCP2*, *GhTCP7a*, *GhTCP8*, *GhTCP9b*, *GhTCP22*, and *GhTCP24*) in early developing ovules/fibers of wild type cotton (cv. Xuzhou142) and its fuzzless-lintless mutant (*fl*). As shown in Fig. 6, *GhTCP8* and *GhTCP22* showed high expression levels in 0–1 DPA *fl* ovules and in –1 DPA Xuzhou 142 ovules. The expression of *GhTCP7a* in Xuzhou 142 ovules was higher than that in *fl* ovules. Interestingly, *GhTCP2* and *GhTCP24* showed opposite expression profiles in ovules of Xuzhou 142 and its *fl* mutant. The expression of *GhTCP2* in –2 to 0 DPA Xuzhou 142 ovules was higher than that in *fl* ovules, while its expression declined in 1 DPA Xuzhou 142 ovules and became lower than that in *fl* ovules. *GhTCP9b* showed relatively high expression activity in –2 DPA Xuzhou 142 ovules, while its expression in −1 to 1 DPA ovules displayed slight difference between Xuzhou 142 and *fl*.

### Interactions among GhTCP proteins and several regulators related to cotton fiber development

**Figure 7:**
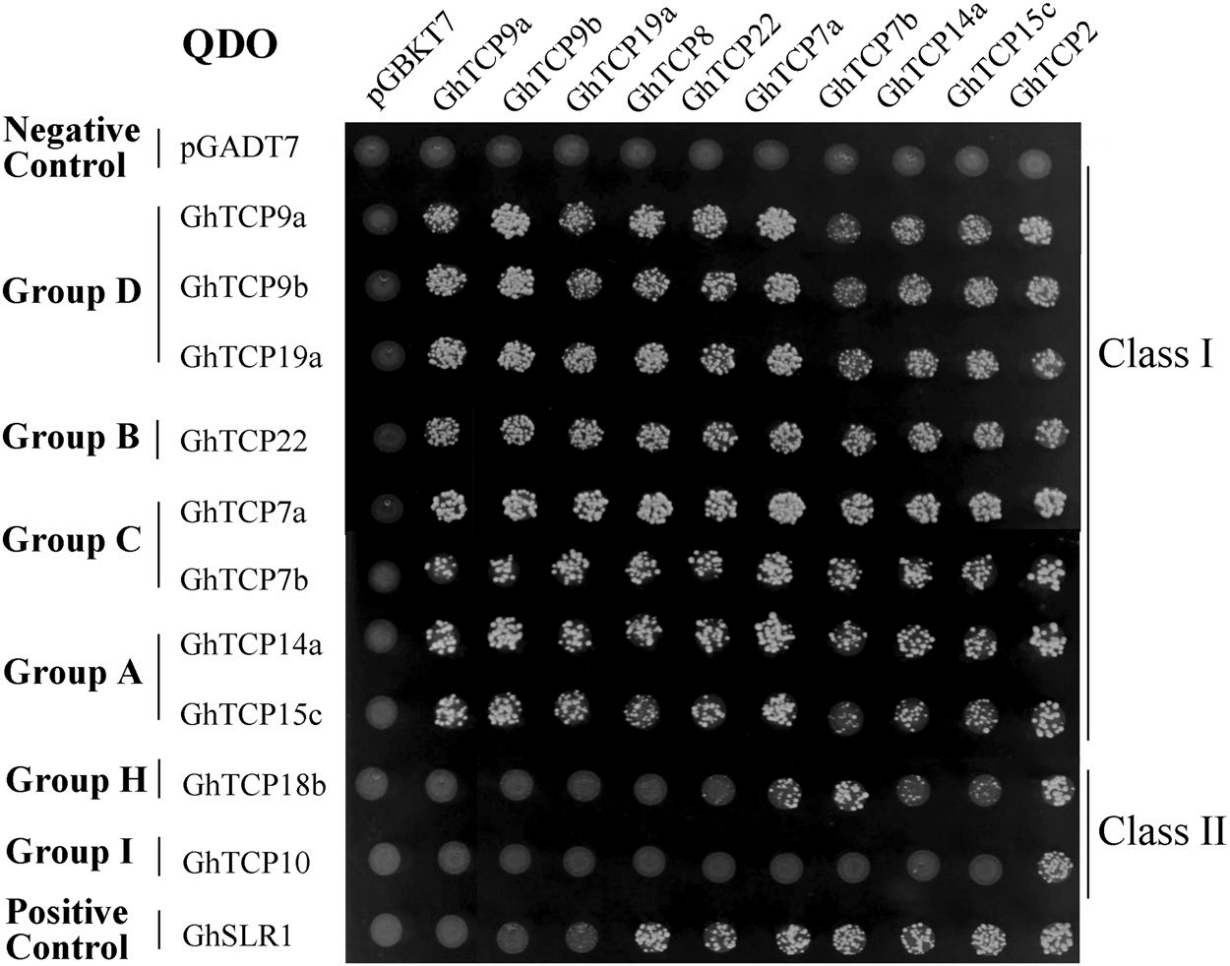
Interactions among GhTCP proteins. Coding sequences of GhTCP genes were cloned into pGADT7 and pGBKT7 vectors. Interactions among the GhTCP proteins were analyzed by yeast two-hybrid assay. Transformants were assayed for growth on QDO nutritional selection medium.

TCP proteins tend to form homodimers or heterodimers that may be required for their DNA-binding activity^3, 9^. To understand how GhTCP proteins interact with each other, yeast two-hybrid technique was employed to analyze the interactions among these GhTCP proteins. The coding sequences of *GhTCP* genes were cloned as translational fusions with the yeast GAL4 TF binding (BD) or activation (AD) domain, and all combinations were tested in a DDO medium (Supplementary Fig. S3). As shown in Fig. 7, all the class I GhTCPs could form both homodimers and heterodimers. GhTCP2, belonging to class II, can interact with all the GhTCPs, while GhTCP18b, another class II TCP, can interact with GhTCP2, GhTCP7a/7b and GhTCP14a/15c. Additionally, GhTCP10 and GhTCP18b have autoactivation activity in yeast on both selection media, while GhTCP22 shows weak autoactivation activity only on TDO medium with 1 mM 3-AT, and group F GhTCPs (GhTCP9a, GhTCP9b and GhTCP19a) can not interact with GhSLR1 (Supplementary Fig. S4).

**Figure 8:**
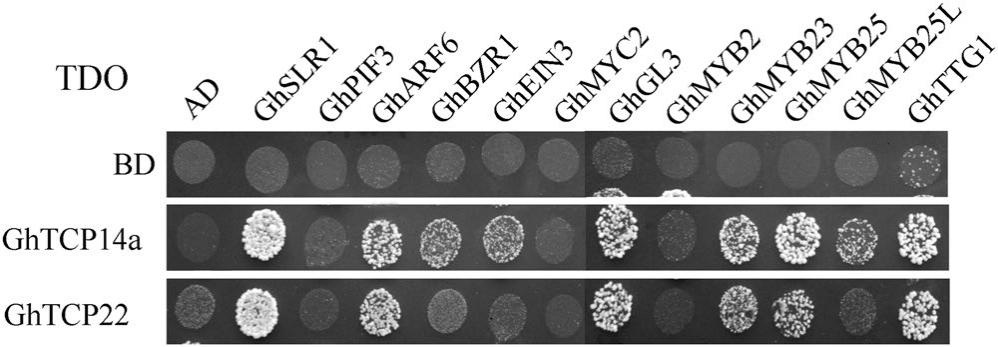
Interactions between GhTCP14a/GhTCP22 and several TFs related to cotton fiber development. Interactions between GhTCP proteins and the TF condidates were analyzed by yeast two-hybrid assay. Transformants were assayed for growth on TDO nutritional selection medium.

We also checked whether GhTCP14a and GhTCP22 can interact with some TFs related to fiber development. As shown in Fig. 8 and Supplementary Fig. S5, GhTCP14a can interact with GhSLR1, GhARF6, GhBZR1, GhEIN3 and GL1-GL3-TTG1 members (GhGL3, GhMYB23, GhMYB25, GhMYB25L and GhTTG1), while GhTCP22a can interact with GhSLR1, GhARF6 and GL1-GL3-TTG1 members (GhGL3, GhMYB23, GhMYB25 and GhTTG1) in yeast cells.

## Discussion

Plant TCP TFs are ancient proteins. The number of TCP proteins is expanded from 5~6 members in pluricellular algae/moss to more than 20 members in Arabidopsis thaliana, rice, and poplar^2, 44, 45^. Recently, genome-wide identification revealed that segmental duplication may be a predominant duplication event for TCP genes and a major contributor to expansion of TCP gene family in two diploid cotton species G. raimondii and G. arboreum^35, 36^. In our study, 74 GhTCP genes were identified in allotetraploid upland cotton genome (AADD). These GhTCPs can be divided into two classes (class I and class II), and class II can be further split into two clades (TB1/CYC clade and CIN clade) (Fig. 3A). TCP domain allows TCP proteins to bind to DNA and to mediate protein-protein interaction^1, 46^. In this study, sequence analysis revealed that TCP domains are highly conserved in each group of GhTCP family, suggesting that the GhTCPs in the same group may share similar DNA binding capacity and protein interaction pattern. Upland cotton TCPs are classified into eleven groups based on their phylogenetic relationship and motif distribution patterns (Figs 1 and 3). GhTCPs members within a sub-clade usually exhibit similar motif composition, while the motif composition among GhTCPs members from distinct clades shows significant difference. Some special motifs are only present in certain clade. Recent studies reported there are about 70,000~76,000 protein-coding genes existing in *G*. *hirsutum* genome^22, 23^, and 27,029 protein-coding genes in *Arabidopsis* genome^37^. This means that there are about 2.6~2.8 times duplication of protein coding genes in the *G*. *hirsutum* genome compared with *Arabidopsis*. Thus, the duplication ratio of *TCP* genes is slightly higher than other gene families in *G*. *hirsutum*. Furthermore, we found the duplication ratio of class I *TCP* genes (3.7 fold) is higher than that of Class II (2.5 fold) during evolution, likely to *G*. *arboretum* and *G*. *raimondii* (Table 2).

Previous studies showed *GhTCP14* (named as *GhTCP14a* in this paper) and *GbTCP* (homolog of *GhTCP15a*) play critical roles in cotton fiber development which are expressed predominantly in initiating and elongating fibers^33, 34^. In our study, *GhTCP14a* and *GhTCP15a* were predominantly expressed in fast elongating fibers (6–12 DPA). In addition, several class I *GhTCPs*, including *GhTCP7a*, *9b*, *15b/c*, *21*, and *22*, were coexpressed with *GhTCP14a* and *GhTCP15a* during cotton fiber development, suggesting that class I *TCPs* may function redundantly in regulating fiber development. Similarly, many class I *TCPs* function redundantly to control plant grow and development in *Arabidopsis* ^8, 15, 41, 43^. Additionally, AtTCP8/14/15/22 interact with DELLA proteins mediating GA signaling^15^. In our study, GhTCP7a, GhTCP14a, GhTCP15a/15b/15c, and GhTCP22 proteins can form homodimer and hetrodimers, and can interact with GhSLR1. These data suggest a GA-regulated DELLA-TCP interaction may also exist in cotton fiber for regulating fiber elongation. The qRT-PCR results also showed several *GhTCPs* were differentially expressed between Xuzhou142 and its natural fuzzless-lintless mutant (*fl*) during cotton fiber initiation (Figs 5C, 6). However, no differentially expressed *GhTCPs* was found in the identified 865 DEGs (differentially expressed genes) between the Xuzhou 142 and *fl* in ovules at −3 and 0 DPA^47^. The reason for this conflict may be that the differential expression levels of the DEGs exhibited in the transcriptome data are over 3 folds^47^, but our results have shown that the differential expression levels of all selected *GhTCPs* genes are less than 3 times between Xuzhou 142 and *fl* ovules (Fig. 6). Additionally, *GhTCP11* is preferentially expressed in fibers at the stage of secondary cell wall biosynthesis, suggesting that this gene may be involved in secondary cell wall formation of fibers. Except that, many *GhTCPs* are preferentially expressed in leaves suggesting these genes may be involved in cotton leaf development, similar to their homologs in *Arabidopsis* ^4, 38–43, 48^. Previous studies showed *CYC/TB1 TCPs* contribute to shoot branching, as well as control the growth and development of axillary buds^2, 49–53^. *Antirrhinum CYC* and *DICH* were expressed in dorsal domain of early floral meristems^49^. *LjCYC2* was expressed in floral meristems and the dorsal organs of developing flowers^52^. *OsTB1* and *AtTCP18* (*AtBRC1*) are expressed in axillary buds^50, 53^. Our results showed that the expression activities of all 8 *G*. *hirsutum CYC/TB1* members (CYC/DICH clade) are very low in the 5 selected cotton tissues (Fig. 3). Hence, their expression patterns in the axillary tissues or developing flowers need to be further investigated.

It has been reported that TCP proteins interact preferentially with those TCP proteins from the same class to form homodimer or heterodimer in *Arabidopsis*, tomato and rice^8, 9^. Similarly, our data revealed that some GhTCP proteins, especially class I TCPs, have the ability to form homodimer and heterodimer. Furthermore, GhTCP10 and GhTCP18b have autoactivation activity, while GhTCP22 showed weak autoactivation in yeast cells (Supplementary Fig. S4). In contrast, other class I GhTCPs did not show any self-activation activities when they were used as baits in yeast two-hybrid assay. Therefore, it is likely that at least some TCP TFs are not transcriptional activators *per se*, and need to interact with other proteins for controlling transcription. Recently, several studies showed that TCPs interact with some TFs, such as DELLAs, AS2, ABI4, MYBs (TT2, PAP1, PAP2, MYB113 and MYB114), and bHLHs (TT8, TOC1), suggesting that TCPs are involved in regulating plant growth and development^11, 13, 15, 16, 18^. Our studies showed GhTCP14a and GhTCP22 interact with GhMYB23/GhMYB25-GhGL3-GhTTG1, the homologs of triplet GL1-GL3-TTG1 that control *Arabidopsis* trichome initiation^27^. GhMYB23/GhMYB25, GhGL3 and GhTTG1 are preferentially expressed in initiating fibers, and promote fiber initiation of cotton^26, 31, 54^. Thus, GhTCP14a and GhTCP22 may play an important role in regulating cotton fiber initiation. Additionally, GhTCP14a and GhTCP22 have the ability to interact with GhSLR1, GhBZR1 and GhARF6. These results suggest that GhTCP14a/22 may participate in controlling cotton fiber elongation via GA, BR and auxin signaling pathways.

In brief, the data presented in this study systematically analyzed *TCP* gene family of upland cotton. Our results lay the foundation for functional characterization of *GhTCP* genes and will lead to further understanding of the structure-function relationship among these *TCP* members. Additionally, our study also provides comprehensive information and novel insights into evolution and divergence of *TCP* genes in upland cotton.

## Materials and Methods

### Plant materials

Upland cotton (*G*. *hirsutum* cv. Coker312, Xuzhou142 and its natural fuzzless-lintless mutant *fl*) seeds were surface sterilized with 70% (v/v) ethanol for 1 min and 10% hydrogen peroxide for 2 h, followed by washing with sterile water. The sterilized seeds were germinated on one-half strength Murashige and Skoog (MS) medium (12-h-light/12-h-dark cycle, 28 °C), and sterile seedlings were transplanted in soil for further growing to maturation. The roots, stems (near the shoot apical meristem) and leaves of four leaves period cotton plants were harvested for RNA extraction. The ovules and cotton fibers in different developmental stage were collected for RNA extraction.

### Identification of GhTCP genes and proteins

The genome sequence of *G*. *hirsutum* was downloaded from the Cotton Genome Project (CGP; http://cgp.genomics.org.cn/page/species/index.jsp) and CottonGen (http://www.cottongen.org/)^22, 23^. In order to identify all members of *TCPs* in *G*. *hirsutum* genome, a BLASTP search was performed against *G*. *hirsutum* protein database in CottonGen using the TCP sequences of *G*. *raimondii* and *G*. *arboreum* as queries. The candidate TCP genes were further aligned to remove redundant sequences. Subsequently, the TCP sequences were manually inspected with MotifScan (http://myhits.isb-sib.ch/cgi-bin/motif_scan) and SMART (http://smart.embl-heidelberg.de/) databases to confirm the presence of the conserved TCP domain. The TCP gene and protein sequences from *Arabidopsis thaliana*, *Theobroma cacao*, *Vitis vinifera*, *Solanum lycopersicum*, *Oryza sativa*, and *Brachypodium distachyon* were retrieved from PlantTFDB plant transcription factor database (http://planttfdb.cbi.pku.edu.cn/), while the GrTCP and GaTCP sequences were obtained from previous studies^35, 36^.

#### DNA and protein sequence analysis

DNA and protein sequences were analyzed using DNASTAR software (DNAStar, MD, USA). Phylogenetic analysis was performed to determine evolutionary relationship among protein sequences. The phylogenetic tree was generated using the Neighbor-Joining (NJ) method implemented in the Clustal X, and output by MEGA 6.06 software (http://www.megasoftware.net/). GhTCP protein sequences were submitted to online Multiple Expectation maximization for Motif Elicitation (MEME) program (http://meme-suite.org/, Version 4.11.0) for identification of conserved protein motifs. The optimized MEME parameters are as follows: any number of repetitions, the optimum width: 6 to 50, maximum number of motifs: 20, and minimum sites per motif: 4.

#### Expression pattern analysis

For the qRT-PCR analysis, total RNA was extracted from roots, stems, leaves, ovules and fibers. RNA was purified using Qiagen RNeasy kit according to the manufacturer’s instructions. First strand of cDNA was reversely synthesized from the purified RNA using Moloney murine leukemia virus reverse transcriptase (Promega) according to the manufacturer’s instructions. Quantative PCR was performed using the fluorescent intercalating dye SYBR-Green (Toyobo) in a detection system (MJ Research; Option 2), and a cotton polyubiquitin gene (*GhUBI1*, GenBank accession no. EU604080) was used as a standard control. A two-step PCR procedure was performed in all experiments using a method described earlier^55^. The relative target gene expression was determined using the comparative cycle threshold method. To achieve optimal amplification, PCR conditions for every primer combination were optimized for annealing temperature and Mg^2+^ concentration. PCR products were confirmed on an agarose gel. Data presented in the qRT-PCR analysis are mean and standard deviation of three biological replicates of plant materials and three technical replicates in each biological sample using gene-specific primers (Supplementary Table 2).

### Heat-map analysis of gene expression

The RPKM (reads per kb per million reads) values denoting the expression levels of *TCP* genes were isolated from a comprehensive profile of the TM-1 transcriptome data (Accession codes, SRA: PRJNA248163)^23, 56^, downloaded from http://www.ncbi.nlm.nih.gov/sra/?term=PRJNA248163. A heat-map analysis was performed using Genesis^57^.

### Yeast two-hybrid assay

The coding sequences of *GhTCP* and TF genes amplified by PCR using Pfu DNA polymerase and gene-specific primers (Supplementary Table 3) were cloned into the different restriction sites of yeast two-hybrid vectors pGBKT7 (bait vector) and pGADT7 (prey vector), creating fusions to the binding domain and activation domain of the yeast transcriptional activator GAL4, respectively. All these constructs were checked by sequencing. The corresponding constructs were co-transformed into Y2HGold yeast strain using the high-efficiency lithium acetate transformation procedure following the manufacturer’s instructions (Clontech). Successfully transformed cell colonies were identified on yeast double drop-out (DDO) medium lacking Leu and Trp after the transformants were incubated on DDO medium at 30 °C for 3–4 days. The positive interactions were identified on yeast quadruple dropouts (QDO) lacking Leu, Trp, His and Ade or on yeast drop-out triple dropouts (TDO) lacking Leu, Trp, and His with 1 mM 3-amino-1,2,4-triazole (3-AT). The pGADT7 empty vector and pGADT7-GhSLR1 were also co-transformed with pGBKT7 constructs as negative and positive controls, respectively.

## Electronic supplementary material

Supporting information
