# Supplementary material for: Genome-wide identification and characterization of TCP transcription factor genes in upland cotton (Gossypium hirsutum)
Source: Sci Rep. 2017 Aug 31;7:10118. doi: 10.1038/s41598-017-10609-2 (PMC5579058; doi:10.1038/s41598-017-10609-2)
Supplement: Supplementary file 1 — Supporting information [file 41598_2017_10609_MOESM1_ESM.pdf]

Genome-wide identification and characterization of TCP transcription factor genes in upland cotton (*Gossypium hirsutum*)

Wen Li[1,+], Deng-Di Li[1,+], Li-Hong Han[1,+], Miao Tao[1], Qian-Qian Hu[1], Wen-Ying Wu[1], Jing-Bo Zhang[1], Xue-Bao Li[1,*], Geng-Qing Huang[1,*]

Supplementary Table 1. Conserved motifs and motif order of GhTCPs.

Subgroup	name	motif order	E value	Class I or II
Group E	GhTCP11-A	1/2/12	3.8e-47	Class I (PCF-like)
	GhTCP11-D	1/2/12	2.5e-48	
Group B	GhTCP8-A	16/1/2/11/9/19/14/18/6	1.0e-130	
	GhTCP8-D	1/2/11/9/19/14/18/6	7.3e-131	
	GhTCP22-A	16/1/2/11/9/19/14/18/6/11	5.1e-203	
	GhTCP22-D	16/1/2/11/9/19/14/18/6/11	3.4e-206	
	GhTCP23-A	11/1/2/11/19/14/18/6/11	1.4e-99	
	GhTCP23-D	11/1/2/11/19/14/18/6/11	1.3e-102	
Group C	GhTCP7a-A	1/2/9/14/12/6	1.0e-82	
	GhTCP7a-D	1/2/9/14/12/6	1.4e-82	
	GhTCP7b-A	18/1/2/9/14/12/6	4.7e-82	
	GhTCP7b-D	18/1/2/9/14/12/6	2.0e-82	
	GhTCP21-A	18/1/2/9/14/12/6	5.9e-76	
	GhTCP21-D	18/1/2/9/14/12/6	1.0e-76	
Group A	GhTCP14a-A	11/9/8/1/2/13/12/10/14/3/6	9.3e-195	
	GhTCP14a-D	11/9/8/1/2/13/12/10/14/3/6	9.3e-195	
	GhTCP14b-A	11/9/8/1/2/13/12/10/14/3/6	1.4e-207	
	GhTCP14b-D	9/8/1/2/13/12/10/14/3/6	8.1e-201	
	GhTCP14c-A	11/9/8/1/2/13/12/10/14/3/6	1.2e-201	
	GhTCP14c-D	9/8/1/2/13/12/10/14/3/6	4.1e-199	
	GhTCP15a-A	9/8/1/2/13/12/10/3/6	4.9e-185	
	GhTCP15a-D	9/8/1/2/13/12/10/3/6	4.9e-185	
	GhTCP15b-A	9/8/1/2/13/18/12/10/14/3/6	3.6e-207	
	GhTCP15b-D	9/8/1/2/13/18/12/10/14/3/6/11	4.8e-212	
	GhTCP15c-A	9/8/1/2/13/18/12/10/14/3/6	5.1e-206	
	GhTCP15c-D	9/8/1/2/13/18/12/10/14/3/6	2.3e-209	
Group F	GhTCP6a-A	18/8/1/2/12/4/5/11	3.4e-192	
	GhTCP6a-D	18/8/1/2/12/4/5/11	1.2e-191	
	GhTCP6b-A	18/8/1/2/12/4/5	2.3e-185	
	GhTCP6b-D	18/8/1/2/12/4/5	3.4e-187	
	GhTCP6c-A	18/8/1/2/12/4/5	7.9e-181	
	GhTCP6c-D	18/8/1/2/12/4/5	6.3e-185	
	GhTCP20a-A	8/1/2/12/4/5/11	7.9e-176	
	GhTCP20a-D	18/8/1/2/12/4/5/11	6.2e-183	
	GhTCP20b-A	18/8/1/2/12/10/5	9.3e-108	
	GhTCP20b-D	18/8/1/2/12/10/5	5.5e-112	
Group D	GhTCP9a-A	8/1/2/18/14/12/9	2.4e-78	
	GhTCP9a-D	8/1/2/18/14/12/9	1.6e-79	
	GhTCP9b-A	1/2/18/14/14/19/9	9.2e-66	
	GhTCP9b-D	1/2/18/14/14/19/9	6.4e-66	
	GhTCP19-A	1/2/18/14/12/9	1.8e-63	
	GhTCP19-D	1/2/18/12/9	6.8e-64	
Group G	GhTCP16-A	1/9	1.2e-35	
	GhTCP16-D	1/9	1.2e-35	
	GhTCP25-A	18/1/20/12/5	1.1e-62	
	GhTCP25-D	18/1/20/12/5	2.9e-62	

Group I	GhTCP3-A	7/1/17/9/11/11/15/11/12	3.7e-134	Class II (CIN-like)
	GhTCP3-D	7/1/17/9/11/11/15/11/12	9.8e-131	
	GhTCP10-A	7/1/17/9/11/11/15/11/12	5.3e-128	
	GhTCP10-D	7/1/17/9/11/11/15/11/12	1.3e-124	
	GhTCP4-A	7/1/17/9/11/15/11/12	2.7e-133	
	GhTCP4-D	7/1/17/9/11/15/11/12	2.1e-133	
Group K	GhTCP5-A	7/1/9/17/12	3.7e-56	
	GhTCP5-D	7/1/9/17/12	9.3e-56	
	GhTCP17-A	12/7/1/11/17	7.6e-50	
	GhTCP17-D	12/7/1/11/17	1.6e-50	
	GhTCP13a-A	12/7/1/10	5.4e-50	
	GhTCP13a-D	12/7/1/10	2.2e-52	
	GhTCP13b-A	12/7/1	9.7e-39	
	GhTCP13b-D	12/7/1	9.9e-38	
Group J	GhTCP2-A	7/1/20/14	1.2e-49	
	GhTCP2-D	7/1/20/14	1.6e-50	
	GhTCP24-A	7/1/20/14/11	2.4e-56	
	GhTCP24-D	7/1/20/14/11	2.4e-56	
Group H	GhTCP1-A	11/1/20	7.9e-44	Class II (CYC/TB1-like)
	GhTCP1-D	11/1/20	2.7e-44	
	GhTCP12-A	18/8/7/1/20	4.6e-41	
	GhTCP12-D	18/8/7/1/20	2.0e-41	
	GhTCP18a-A	10/1/9/20/11	7.6e-45	
	GhTCP18a-D	1/9/20	2.3e-43	
	GhTCP18b-A	1/11/12/1/19/20/11/13	5.6e-58	
	GhTCP18b-D	9/11/12/1/20/11	4.3e-54	

Supplementary Table 2. The RPKM (reads per kb per million reads) values denoting the expression levels of *TCP* genes.

Gene name	Gene symbol	-3ovule	0-ovule	3-ovule	5dpa	10dpa	20dpa	25dpa
GhTCP1-A	Gh_A07G1572	0	0	0	0	0	0	0.058043
GhTCP2-A	Gh_A05G1236	43.128	39.1682	38.97949	34.831	24.0063	28.218	11.5611
GhTCP2-D	Gh_D05G3838	38.2179	31.6293	27.87133	21.2901	15.3152	24.3422	10.3497
GhTCP3-A	Gh_A01G0414	3.793684	4.134738	5.055131	2.952592	2.295177	1.255306	13.5947
GhTCP3-D	Gh_D01G0419	3.718353	5.239975	3.970553	2.521547	1.675268	4.493948	16.8245
GhTCP4-A	Gh_A04G0316	2.911143	0.860544	3.658502	4.807865	6.048547	3.853258	6.051921
GhTCP4-D	Gh_D05G3332	2.144128	1.185044	2.18743	1.422124	2.374428	5.526826	8.007604
GhTCP5-A	Gh_A12G1657	0.12121	0	0.023253	0	0.027479	0	1.332641
GhTCP5-D	Gh_D12G1814	0.898554	0.02851	0.359589	0	0.081935	0.75718	1.923578
GhTCP6a-A	Gh_A10G0634	1.092935	1.057684	2.195825	1.017906	1.426371	4.477338	2.761232
GhTCP6a-D	Gh_D10G0762	1.542673	0.51461	1.735965	0.746674	1.020341	2.682712	2.006759
GhTCP6b-A	Gh_A05G2936	0.90832	1.6785	1.346253	1.32003	0.83765	24.8497	13.3814
GhTCP6b-D	Gh_D04G0721	2.89942	3.02401	2.940257	1.27074	0.86977	40.7474	21.0792
GhTCP6c-A	Gh_A09G2496	0.596217	0.29071	0.264979	0.323893	0.029961	1.038274	0.792578
GhTCP6c-D	Gh_D09G0381	0.248791	0.361026	0.514521	0.51553	0.382072	1.610524	0.862666
GhTCP7a-A	Gh_A03G1464	4.119916	5.382942	10.66243	13.0509	3.944947	2.565243	4.469166
GhTCP7a-D	Gh_D02G1925	4.31742	6.696104	10.86611	18.3383	8.439013	2.190774	3.890616
GhTCP7b-A	Gh_A13G0528	9.595801	7.413611	10.68479	6.562331	7.867909	1.891182	3.83915
GhTCP7b-D	Gh_D13G0602	12.12343	8.324929	11.82406	7.800414	6.28633	1.395857	2.821856
GhTCP8-A	Gh_A04G1120	8.93018	3.92743	4.634318	3.11299	1.34818	5.2084	4.10954
GhTCP8-D	Gh_D04G1732	9.87358	5.52163	5.85813	3.04966	1.55976	6.91149	4.39986
GhTCP9a-A	Gh_A11G0759	12.17658	4.72164	7.297966	5.002134	3.354901	4.691452	6.603302
GhTCP9a-D	Gh_D11G0887	11.33406	5.819478	6.712125	3.527974	2.438645	3.954006	4.643692
GhTCP9b-A	Gh_A12G2051	38.1998	12.8067	14.15322	4.520842	2.31353	0.513792	1.025389
GhTCP9b-D	Gh_D12G2229	22.0801	18.1542	13.06758	5.74622	4.009062	0.550683	0.469307
GhTCP10-A	Gh_A13G1272	10.633	9.87016	9.646767	9.55502	8.91926	9.21566	12.3753
GhTCP10-D	Gh_D13G1576	9.49409	8.5764	9.267314	9.58347	9.66616	10.8616	12.0864
GhTCP11-A	Gh_A09G1389	1.07687	0.959679	3.096471	1.797841	1.611111	7.681179	4.844826
GhTCP11-D	Gh_D09G1394	1.756258	1.166791	1.192285	1.006552	2.253806	4.8011	4.903123
GhTCP12-A	Gh_A12G1561	0.154037	0	0.182439	0.049878	0	0.027485	0.099992
GhTCP12-D	Gh_D12G1689	0.054919	0	0.013568	0.024898	0	0.027485	0.133322
GhTCP13a-A	Gh_A05G3219	2.304494	1.878745	1.674126	0.409191	1.226476	5.375785	3.454591
GhTCP13a-D	Gh_D04G0387	4.002478	2.956107	3.021519	1.045731	2.590998	6.718204	6.34285
GhTCP13b-A	Gh_A09G0084	9.204154	6.644285	6.950844	6.14395	1.981187	4.46023	4.077899
GhTCP14a-A	Gh_A11G0279	8.64517	7.04354	19.34541	28.3725	37.5771	0.69167	1.39015
GhTCP14a-D	Gh_D11G0333	10.1867	6.74463	19.76992	24.0844	31.0267	0.53488	1.14207
GhTCP14b-A	Gh_A07G0574	8.45262	5.64745	4.567338	3.42401	2.22818	0.64115	0.68934
GhTCP14b-D	Gh_D07G0639	17.334	16.5035	11.14922	4.28985	1.95995	2.44597	1.46268
GhTCP14c-A	Gh_A12G1603	5.60733	1.74658	1.219029	0.74948	0.03603	0.2373	0.08019
GhTCP14c-D	Gh_D12G1742	6.78435	2.04705	2.780272	1.37901	0.05957	0.24085	0.18756
GhTCP15a-A	Gh_A12G1522	0.17479	1.42741	1.004499	1.6413	22.5323	0	0.40808

GhTCP15a-D	Gh_D12G1644	0.37823	0.88895	1.166681	4.36272	31.3828	0	0.90963
GhTCP15b-A	Gh_A13G0648	2.14995	3.96098	7.045882	2.1475	9.56593	0.23199	0.42554
GhTCP15b-D	Gh_D13G2530	4.14437	4.99955	13.83429	4.33449	11.7877	4.00236	3.16691
GhTCP15c-A	Gh_A13G0647	6.76533	15.2444	10.92444	8.12662	18.4899	6.1579	2.60161
GhTCP15c-D	Gh_D13G2529	3.76945	8.28306	21.74904	2.13316	9.61785	0.59557	1.299
GhTCP16-A	Gh_A13G2021	1.926086	2.25553	1.303538	0.523737	0.633924	1.214918	0.632378
GhTCP16-D	Gh_D13G2419	0.91579	1.722709	0.954937	0.364914	0.180993	0.420229	0.459988
GhTCP17-A	Gh_A07G0613	0	0	0	0	0	0.077514	0.141879
GhTCP17-D	Gh_D07G0680	0	0	0	0	0	0.077514	0.020889
GhTCP18a-A	Gh_A11G0057	0	0	0	0	0	0	0
GhTCP18a-D	Gh_D11G0061	0	0	0	0	0	0	0
GhTCP18b-A	Gh_A12G2405	0.045219	0.588231	0.108298	0.198385	0.09689	0	0.045467
GhTCP18b-D	Gh_D12G2641	0	0.014592	0	0	0	0	0
GhTCP19a-A	Gh_A09G1605	10.3525	13.894	9.2614	1.56312	2.00991	9.98723	7.80271
GhTCP19a-D	Gh_D09G1703	11.3708	9.36467	9.515641	1.4661	1.60675	6.75467	6.98464
GhTCP19b-A	Gh_A08G1602	1.173022	0.826524	0.70926	0.22393	0.352036	0.436173	0.357753
GhTCP19b-D	Gh_D08G1913	1.111985	0.656406	4.163317	0.252796	0.242919	0.537478	0.296383
GhTCP20a-A	Gh_A07G2121	6.263062	4.025104	0.734858	1.492104	0.667754	2.173096	3.078708
GhTCP20a-D	Gh_D07G2330	2.192435	3.880327	0.955892	1.622325	0.840964	3.545393	3.756142
GhTCP20b-A	Gh_A12G1302	7.46456	4.23114	4.475074	27.2127	38.5913	19.0614	13.2008
GhTCP20b-D	Gh_D12G1425	6.11779	5.25935	2.813079	11.1875	16.479	6.61042	5.37929
GhTCP21-A	Gh_A12G1214	23.0958	12.6982	11.52413	12.5544	14.6172	3.73729	6.56445
GhTCP21-D	Gh_D12G1337	23.0359	13.0287	6.654194	17.7348	33.6284	3.14037	6.75964
GhTCP22-A	Gh_A01G1534	0.716273	2.570905	4.777318	11.73482	14.83804	4.118777	2.494799
GhTCP22-D	Gh_D01G1783	1.714007	1.13131	1.668551	5.328265	14.22278	0.368229	0.374377
GhTCP23-A	Gh_A05G2343	48.9062	37.4553	9.064555	22.04	15.6365	16.506	13.8017
GhTCP23-D	Gh_D05G2610	5.764909	5.616406	3.47111	1.732839	1.09635	3.167462	1.337021
GhTCP24-A	Gh_A10G0394	2.774961	2.571037	3.232758	0.475033	1.329804	0.330611	0.089447
GhTCP25-D	Gh_D04G0925	2.453037	0.959975	3.27916	2.000638	1.077513	1.096347	0.667987
GhTCP25-A	Gh_A04G0489	5.559862	5.486126	0.680305	2.723046	12.3825	2.415755	2.801447

Supplementary Table 3. Primers used in this study.

Primer name	Sequence 5’-3’	purpose
GhTCP1 qRT-up	TCGAGGAATCTCTAGTGATCAG	qRT-PCR
GhTCP1 qRT-dn	CCATACAGTTGAACTCCTGTTG	qRT-PCR
GhTCP2 qRT-up	GATACCACCATTTACTGTGTCG	qRT-PCR
GhTCP2 qRT-dn	GGTGGTGATCCCTCTATAGAAG	qRT-PCR
GhTCP3 qRT-up	GTTCGTGCTTGGATAGACCAG	qRT-PCR
GhTCP3 qRT-dn	GCTTATTGGCAATGCTGTCGTG	qRT-PCR
GhTCP4 qRT-up	GCAACAGTCATTGCCACTGACT	qRT-PCR
GhTCP4 qRT-dn	CTCTTGGCCCTGAATTCGTGAC	qRT-PCR
GhTCP5 qRT-up	TGTCTCAGTTTGGAAACCATG	qRT-PCR
GhTCP5 qRT-dn	GTGTATGAGTGAGGCCAGCGT	qRT-PCR
GhTCP6a qRT-up	GCAGTGAAGGTTCTAATTATCTT	qRT-PCR
GhTCP6a qRT-dn	TTGCTGTTGTTGATGCACTCTC	qRT-PCR
GhTCP6b qRT-up	GCAGTGATAGTTCCAATTATCTG	qRT-PCR
GhTCP6b qRT-dn	CTGATGTTGGGGTTGCATCCGA	qRT-PCR
GhTCP6c qRT-up	GCACTGAAAGTCCTATTTATCTG	qRT-PCR
GhTCP6c qRT-dn	CTGTTGCTGGAGCACCCTAGC	qRT-PCR
GhTCP7a qRT-up	GCCAGACTTTGGTCAATTGTG	qRT-PCR
GhTCP7a qRT-dn	GACAACGAGGCTAACAAGTTTAG	qRT-PCR
GhTCP7b qRT-up	GGCTCAGCAACCAGCTGCAGC	qRT-PCR
GhTCP7b qRT-dn	TCAACGAGGGTCATCGTCTCTT	qRT-PCR
GhTCP8 qRT-up	CTAGAGTCCCAAGGTGGTAGAG	qRT-PCR
GhTCP8 qRT-dn	GTACCTTGTTGTTGATGATGCTC	qRT-PCR
GhTCP9a qRT-up	TCACATTCCCAGCCACAGCTAC	qRT-PCR
GhTCP9a qRT-dn	CTCTGAGCATCTGAGTCGTTGT	qRT-PCR
GhTCP9b qRT-up	CTCAAGCTGTGGTGTGCGACGG	qRT-PCR
GhTCP9b qRT-dn	GACCCATCAACTGCAACTCTTG	qRT-PCR
GhTCP10 qRT-up	TCAGAGGGGACCCCTTCAGTC	qRT-PCR
GhTCP10 qRT-dn	GAAGCAGAGGACAGCTTATTTG	qRT-PCR
GhTCP11 qRT-up	AGTTGCCGGCTGGACTTGTGC	qRT-PCR
GhTCP11 qRT-dn	GACCTAGGTAGTGGCTGCTCG	qRT-PCR
GhTCP12 qRT-up	TCATGACGGACCCTAATGAGC	qRT-PCR
GhTCP12 qRT-dn	CGTGGTAATCTGAATGCAAGCC	qRT-PCR
GhTCP13a qRT-up	GACTGAAGAAGTACACAATTTC	qRT-PCR
GhTCP13a qRT-dn	ACCAGGCTGACTTCCATTGATG	qRT-PCR
GhTCP13b qRT-up	TCAACGTTGTGGCAGCACCAT	qRT-PCR
GhTCP13b qRT-dn	GAGGCATCAATTGGAAGGGTC	qRT-PCR
GhTCP14a qRT-up	GGAACTATGTCTAGTGGGTTGC	qRT-PCR
GhTCP14a qRT-dn	CGTGGTGTGAATGTGAGCCAC	qRT-PCR
GhTCP14b qRT-up	CATGAATTTTCCAGCTCCGATG	qRT-PCR
GhTCP14b qRT-dn	GACTAGTTGCATCATGTCGGTT	qRT-PCR
GhTCP14c qRT-up	TCAGTTACTAACAGTGCTTTGT	qRT-PCR
GhTCP14c qRT-dn	GACTAGTTGTATCATGTCGATC	qRT-PCR
GhTCP15a qRT-up	TACCACGTCTAGTGGGGTGCAT	qRT-PCR
GhTCP15a qRT-dn	GCTACTTGAATCATGCCTATGT	qRT-PCR
GhTCP15b qRT-up	CGGCAACCAAGTCATCAGTG	qRT-PCR
GhTCP15b qRT-dn	GTCTAGTGACGCCAGCATACTT	qRT-PCR
GhTCP15c qRT-up	TGGCAACCAAGTTATCAGTGG	qRT-PCR
GhTCP15c qRT-dn	ATTTAGTGACGCCAGCATGCTT	qRT-PCR
GhTCP16 qRT-up	CAACAACAACAGCACCAACACT	qRT-PCR
GhTCP16 qRT-dn	ATGATTTGCAGCTGCTGCCAC	qRT-PCR

GhTCP17 qRT-up	GTTCAACACTGGGACTAATTC	qRT-PCR
GhTCP17 qRT-dn	CTGATCACCTGCAGCATCTTG	qRT-PCR
GhTCP18a qRT-up	GTCCTCTCGCAAGGGATATGAG	qRT-PCR
GhTCP18a qRT-dn	TCCAGTGTTTTGCAGACAATTC	qRT-PCR
GhTCP18b qRT-up	CCAAACTCAGTTCATGGTGGTC	qRT-PCR
GhTCP18b qRT-dn	CCAAAGGGCAGAAAGCCAGTG	qRT-PCR
GhTCP19a qRT-up	GCTGCACCTGGTTCAAACCAG	qRT-PCR
GhTCP19a qRT-dn	CTAAGCATCTGCGTAGTACTAT	qRT-PCR
GhTCP19b qRT-up	GCAAGACCCATATCTTCAATC	qRT-PCR
GhTCP19b qRT-dn	CTTGCATCTGCAGCCTGATGA	qRT-PCR
GhTCP20a qRT-up	GCACTGAAAGTTCAAATTATCTG	qRT-PCR
GhTCP20a qRT-dn	GTGCTGGTGCTGGTGCTGGAAC	qRT-PCR
GhTCP20b qRT-up	TGTGAGTTTTTCGTCGCTGCTC	qRT-PCR
GhTCP20b qRT-dn	CTTATCAGCAACAATCTGCTGC	qRT-PCR
GhTCP21 qRT-up	GTTCAGCAACAGCAAGCGATG	qRT-PCR
GhTCP21 qRT-dn	CTCTTCCACATCCACACACATC	qRT-PCR
GhTCP22 qRT-up	TGAATTCCAAGGTGGTAGAGCT	qRT-PCR
GhTCP22 qRT-dn	TCTCCACTGTCTGTACCTTGAG	qRT-PCR
GhTCP23 qRT-up	GTGCCAGAAGCCCAGATGTG	qRT-PCR
GhTCP23 qRT-dn	TTCATGATGATGCTGCTGTTCC	qRT-PCR
GhTCP24 qRT-up	TGACTACAACCTCAATTTCAC	qRT-PCR
GhTCP24 qRT-dn	GTCTGAGCTCCTGTTTCCATC	qRT-PCR
GhTCP25 qRT-up	GCATAGACGGAGATTCAAACAG	qRT-PCR
GhTCP25 qRT-dn	TTGCAGACCAACCAACAGAATC	qRT-PCR
GhUBI1 qRT-up	CTGAATCTTCGCTTTCACGTTATC	qRT-PCR
GhUBI1 RT-dn	GGGATGCAAATCTTCGTGAAAAC	qRT-PCR
GhTCP2 EcoRI-F	CCGGAATTCATGGAGATAGATGAGATTCAGAC	Y2H
GhTCP2 BamHI-R	CGCGGATCCATGGTGGTGATGGTTCTCCACTG	Y2H
GhTCP7a NdeI-F	CTTCATATGTCCAATTCAGAGACTGCTGCTAC	Y2H
GhTCP7a XmaI-R	CCCCCCGGGATGCGGATCCTCGTCTCTTCGAC	Y2H
GhTCP7b NdeI-F	CTTCATATGTCAAATTCAGAGGCTGCTGCTAA	Y2H
GhTCP7b XmaI-R	CCCCCCGGGACGAGGGTCATCGTCTCTTTGAC	Y2H
GhTCP8 NdeI-F	CTTCATATGGAACTTACATATTCTCAGAACAAT	Y2H
GhTCP8 XmaI-R	CCCCCCGGGTTGAGAATTATTTGGGTTTTCATC	Y2H
GhTCP9a NdeI-F	CTTCATATGGCTGCAATTCATAAACTTGAGGT	Y2H
GhTCP9a XmaI-R	CCCCCCGGGATGCTTTGAAGATCGAGTCATG	Y2H
GhTCP9b NdeI-F	CTTCATATGGATCCTTCTGAAGACGGAGGC	Y2H
GhTCP9b XmaI-R	CCCCCCGGGTTGCTTAGAAGCAGCTTTTATCTG	Y2H
GhTCP10 NdeI-F	CTTCATATGGGAGAAAGCAATCACCAAGCTGC	Y2H
GhTCP10 XmaI-R	CCCCCCGGGATGGTGAGAGTCAGAGGAAGCAG	Y2H
GhTCP14a NdeI-F	CTTCATATGGAAGGAGGAGGCAGTGATGATCAT	Y2H
GhTCP14a XmaI-R	CCCCCCGGGTTCATCGCCGCCACCTCCACCAC	Y2H
GhTCP15c NdeI-F	CTTCATATGATCATGGATGAAAATGGGATCCG	Y2H
GhTCP15c XmaI-R	CCCCCCGGGGCTAGTTGAATCATGCCTATTTTC	Y2H
GhTCP18b NdeI-F	CTTCATATGTATCCTTCAAACAGCAATGGCAG	Y2H
GhTCP18b XmaI-R	CCCCCCGGGAATTTTATTGTTGTAGCCTTCCCA	Y2H
GhTCP19 NdeI-F	CTTCATATGGAGTGCAATCGATATCAAACGAT	Y2H
GhTCP19 XmaI-R	CCCCCCGGGAGGTTTCGAACATGGGGCTTGC	Y2H
GhTCP22 NdeI-F	CTTCATATGGAACTTACAGATTTGCAAAGCAAT	Y2H
GhTCP22 XmaI-R	CCCCCCGGGTTGAGAAGTATTAGGGTCTTGGTC	Y2H
GhSLR1 NdeI-F	CTTCATATGGACGACGAGTTATTAGCTGTT	Y2H
GhSLR1 XmaI-R	CCCCCCGGGACTCAGCTCCTGAGTTAACTC	Y2H
GhPIF3 EcoRI-F	CCGGAATTCATGCCTCTATCCGAGCCATATC	Y2H

GhPIF3 BamHI-R	CGCGGATCCTCACCTATCAGGTAACTTCTT	Y2H
GhARF6 NdeI-F	CGCCATATGAGGCTTTCTTCAGCTGGT	Y2H
GhARF6 XmaI-R	CCCCCCGGGTCAGTAGTCCAATGACCCCAC	Y2H
GhBZR1 EcoRI-F	CTTGAATTCATGACGTCAGATGGGGCGAC	Y2H
GhBZR1 BamHI-R	CTTGGATCCTCAACATCGAGCTTTCCCAC	Y2H
GhEIN3 BamHI-F	CTTGGATCCACATGATGATGTTCGATGAGATG	Y2H
GhEIN3 XhoI-R	CTTCTCGAGTCAAAACCAGATTGAAACATCCTG	Y2H
GhMYC2 NdeI-F	CCGCATATGacggactatcagttagcac	Y2H
GhMYC2 XmaI-R	CCCCCCGGGtcttgcatctccaagtttggttg	Y2H
GhDEL65 BamHI-F	CTTGGATCCACATGTCTACTGGAGTTCAACATC	Y2H
GhDEL65 XhoI-R	CTTCTCGAGTCAACACTTGCTAGCAATTCTT	Y2H
GhMYB2 NdeI-F	CTTCATATGatggctccaaagaaggctggag	Y2H
GhMYB2 XhoI-R	CTTCTCGAGttataccattgctaatggatcct	Y2H
GhMYB23 NdeI-F	CTTCATATGGAAGAAAAGAGTGAATATAA	Y2H
GhMYB23 SacI-R	CTTGAGCTCTTAGGCAAAGCCATGCCAAAC	Y2H
GhMYB25 NdeI-F	CTTCATATGatggggagatcaccatgttgtg	Y2H
GhMYB25 BamHI-R	CTTGGATCCtcataaaccattaaatccaaac	Y2H
GhMYB25L NdeI-F	CTTCATATGatgcagcagtctccatgtagcg	Y2H
GhMYB25L BamHI-R	CTTGGATCCtcaaaagacagaagaaccagatg	Y2H
GhTTG1 NdeI-F	CTTCATATGatggagaattcaactcaagaatcc	Y2H
GhTTG1 BamHI-R	CTTGGATCCtcaaactttgagaagctgcaatt	Y2H

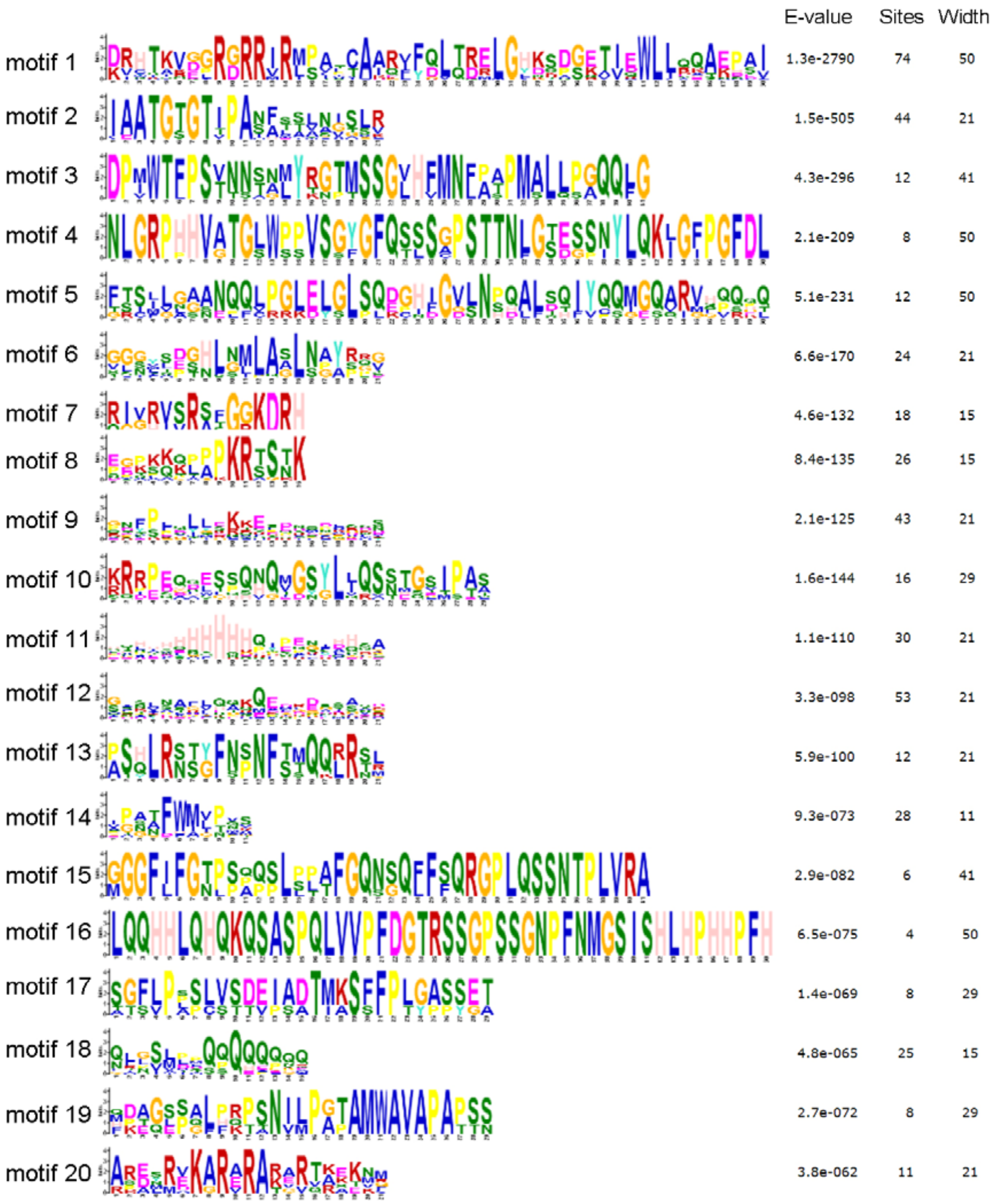

Supplementary Figure 1. Sequence logo of the motifs of GhTCPs.

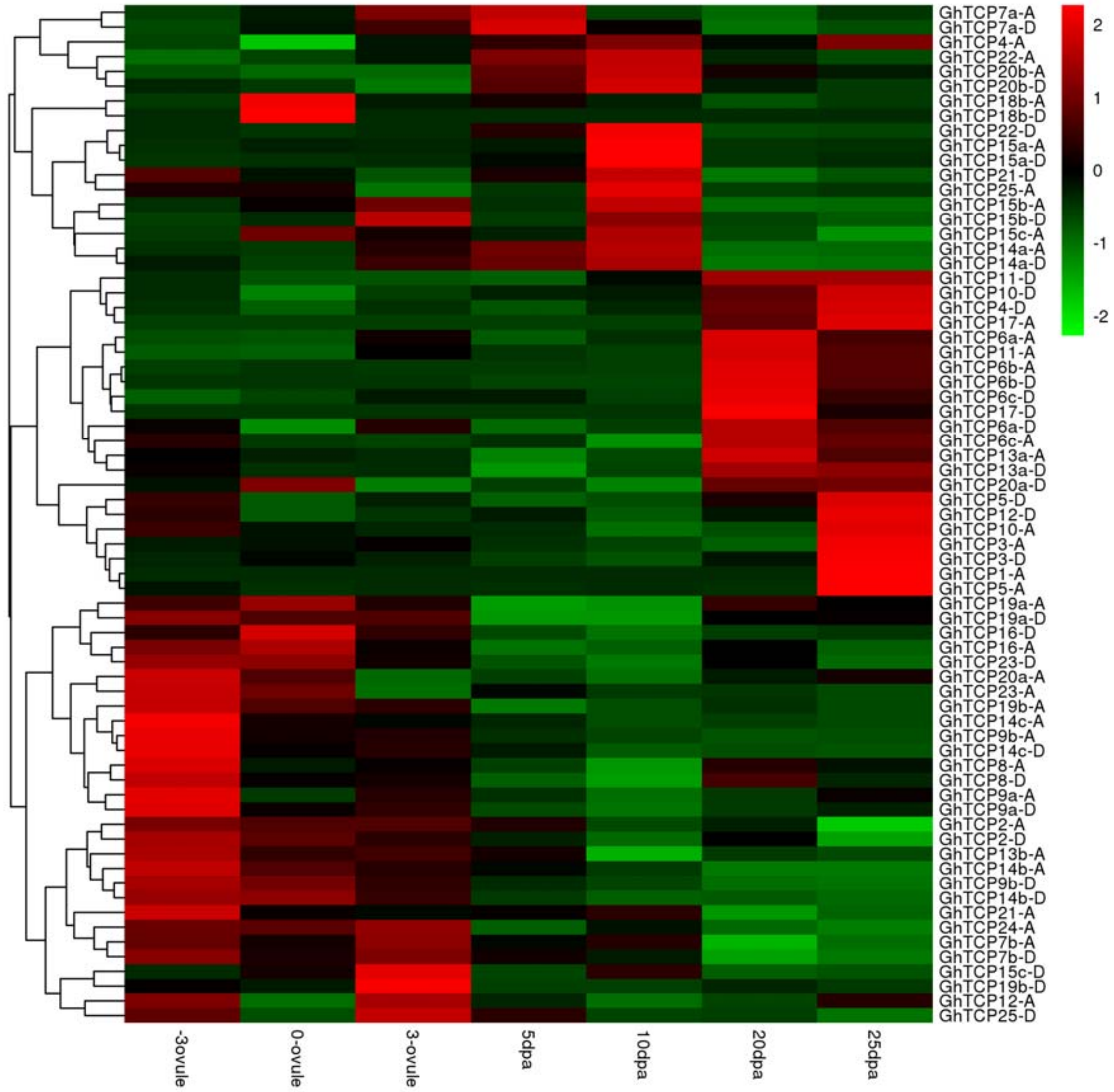

Supplementary Figure 2. Expression heat map of cotton *TCP* genes during cotton fiber development. Expression values are log2-transformed

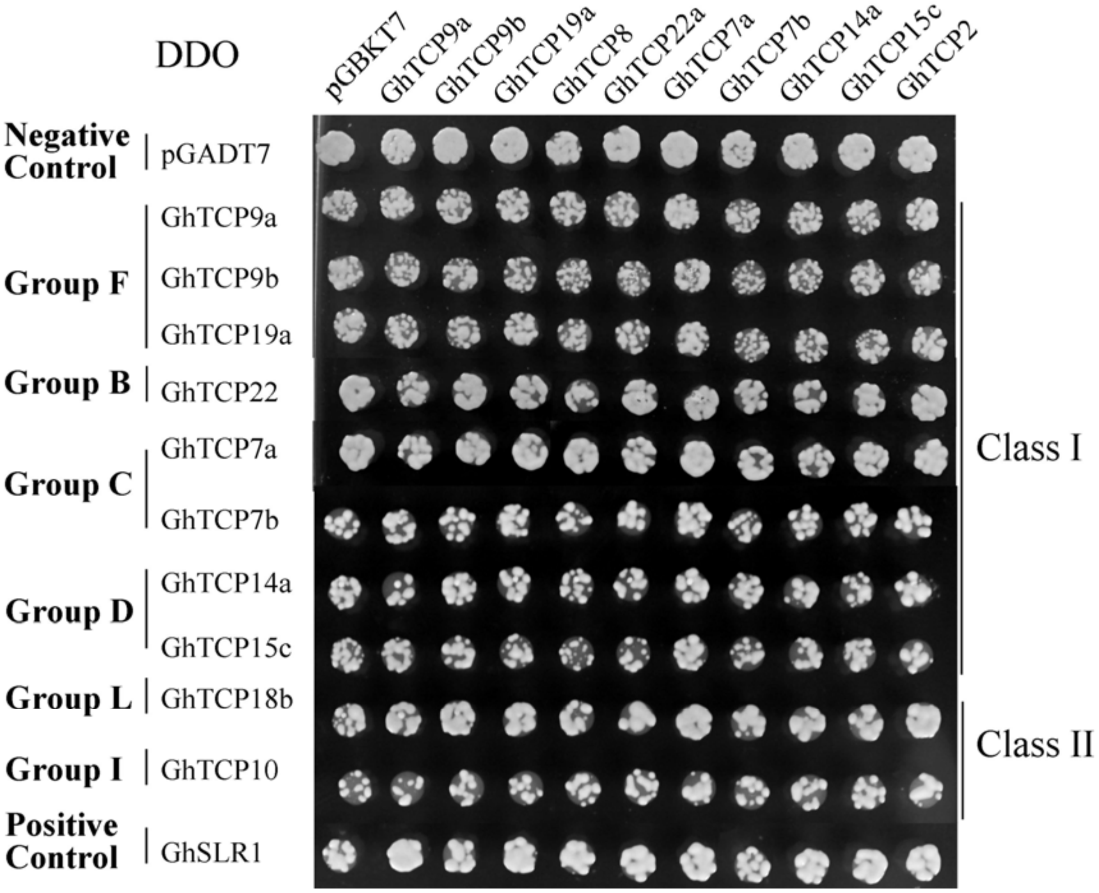

Supplementary Figure 3. Interaction among GhTCPs in yeast cells grown on DDO medium.

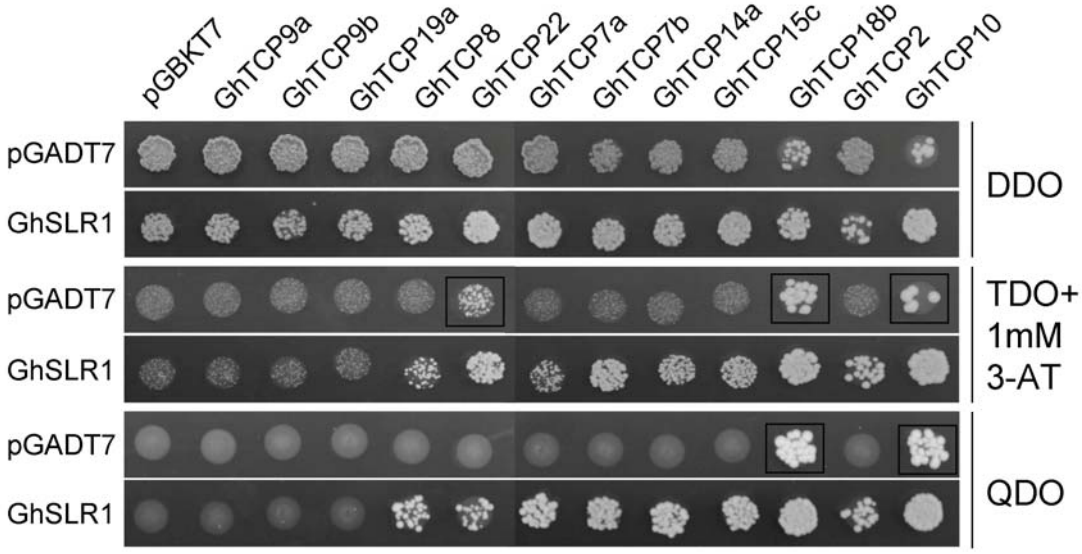

Supplementary Figure 4. Transcriptional autoactivation analysis of GhTCPs in yeast cells.

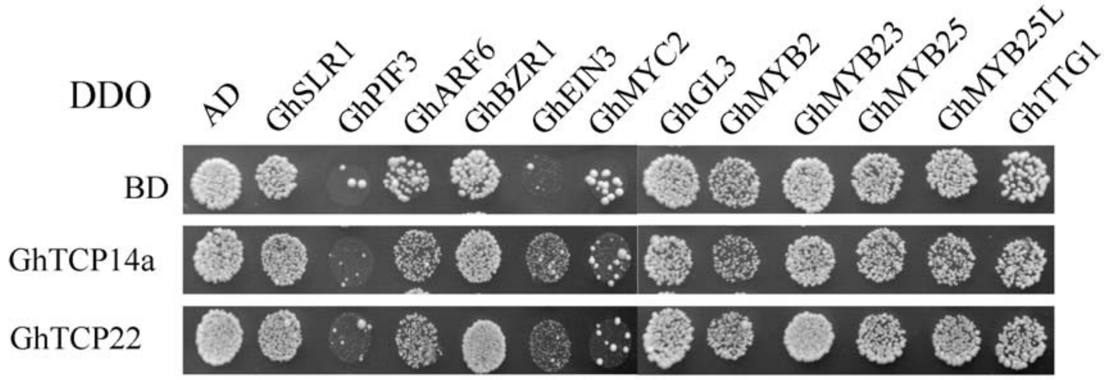

Supplementary Figure 5. Interaction between GhTCPs and some TFs related to cotton fiber development in yeast cells grown on DDO medium.
